# PRDX5 and PRDX6 translocation and oligomerization in bull sperm: a response to cryopreservation-induced oxidative stress

**DOI:** 10.1186/s12964-024-02015-9

**Published:** 2025-01-09

**Authors:** Mostek-Majewska Agnieszka, Bossowska-Nowicka Magdalena, Słowińska Mariola, Ciereszko Andrzej

**Affiliations:** Institute of Animal Reproduction and Food Research, Olsztyn, Poland

**Keywords:** Bull sperm, PRDX5, PRDX6, Oxidative stress, Cryopreservation

## Abstract

Cryopreservation of bull sperm, crucial for breeding and assisted reproduction, often reduces sperm quality due to oxidative stress. This study examines how oxidative stress during cryopreservation affects peroxiredoxin 5 (PRDX5) and peroxiredoxin 6 (PRDX6) proteins, leading to their translocation and oligomerization in bull sperm. Increased reactive oxygen species (ROS) and nitric oxide (NO) levels were linked to reduced mitochondrial potential, higher DNA fragmentation, and increased membrane fluidity, prompting PRDX5 to move intracellularly and PRDX6 to the cell membrane. Under cryopreservation, these proteins formed high molecular weight oligomers, that may shift from peroxidase to chaperone roles. Their interaction with Toll-like receptor 4 (TLR4) may be key to their intracellular transport. On the other hand, the presence of PRDX5 and PRDX6 in exosomal vesicles suggested a potential mechanism for their transport into sperm cells. Using Imaging Flow Cytometry and various PAGE techniques, the study detected PRDX5 and PRDX6 in different sperm locations and analyzed their oligomer formation. These findings highlight the adaptive roles of PRDX5 and PRDX6 in protecting sperm cells, offering insights that could improve cryopreservation protocols in animal breeding and human reproductive medicine, and advance our understanding of the oxidative stress response in sperm cells.

**Supplementary Information:**

The online version contains supplementary material available at 10.1186/s12964-024-02015-9.

## Background

Cryopreservation of bull sperm is a cornerstone of animal breeding and reproductive technologies, but it often compromises sperm quality due to oxidative stress. This process generates reactive oxygen species (ROS), which induce DNA fragmentation, reduce mitochondrial potential, and alter membrane fluidity. These changes impair sperm motility and viability while triggering the translocation and oligomerization of antioxidant proteins that are crucial for protecting sperm from oxidative damage [1, 2]. The imbalance between ROS production and antioxidant defenses is a major cause of this damage, leading to lipid peroxidation, DNA damage, and further reduction in motility. Sperm are particularly vulnerable to oxidative stress because their membranes are rich in polyunsaturated fatty acids and their cytoplasm has limited antioxidant enzyme reserves [3, 4].

To counteract oxidative stress, peroxiredoxins (PRDXs) are vital in safeguarding sperm, making them integral to male fertility. Among the six known PRDX isoforms, PRDX5 and PRDX6 play distinct but complementary roles. PRDX5, an atypical 2-Cys peroxiredoxin, protects intracellular components by scavenging ROS, particularly within the mitochondrial sheath, thereby supporting mitochondrial function, motility, and viability [5]. PRDX6, a 1-Cys peroxiredoxin, focuses on plasma membrane protection. Its dual roles as a peroxidase and a phospholipase A2 enable it to repair oxidized phospholipids, preserving membrane fluidity and sperm’s fertilization capacity [6]. Our previous research has shown that inhibiting PRDX activity exacerbates oxidative damage to mitochondrial and cytoskeletal proteins, while active PRDXs protect key enzymes in energy production and the respiratory chain under stress [7]. Additionally, PRDXs influence post-translational modifications like protein phosphorylation, S-glutathionylation, and S-nitrosylation, which are essential for maintaining redox balance and supporting capacitation [8].

Under conditions of severe oxidative stress, PRDX5 and PRDX6 shift from their typical enzymatic roles to chaperone-like functions by forming high-molecular-weight oligomers. This adaptive mechanism enhances resistance to oxidative damage, as observed in somatic cells. For instance, PRDX5 forms intermolecular disulfide bonds in cardiac tissues to combat oxidative stress, while PRDX6 undergoes structural changes to prevent neuronal apoptosis and manage endoplasmic reticulum stress in lung epithelial cells [9–11]. These findings underscore the versatility of PRDXs in protecting cells under extreme conditions. However, the formation and significance of PRDX complexes in sperm cells remain largely unexplored, emphasizing the need for further research in this area.

Another layer of oxidative stress management in sperm involves Toll-like receptor 4 (TLR4), a key component of the innate immune system. TLR4 not only detects pathogens and triggers inflammatory responses through the NF-κB pathway [12] but also modulates oxidative stress responses in sperm. Recent studies reveal that TLR4 interacts with PRDX5 and PRDX6, enhancing their protective roles. PRDX5’s interaction with TLR4 can trigger pro-inflammatory responses that may help manage oxidative stress, while PRDX6’s enzymatic activities, facilitated by TLR4, help maintain cellular homeostasis during stress conditions [13–15]. This interplay between TLR4 and PRDXs ensures the proper localization and function of antioxidant enzymes, preserving sperm viability and enhancing their fertilization potential.

The aim of this study is to explore the role of cryopreservation-induced oxidative stress in the translocation and oligomerization of PRDX5 and PRDX6 proteins in bull sperm. Specifically, the research seeks to understand how these proteins adapt to oxidative conditions by shifting from their typical peroxidase activity to chaperone roles and their impact on thawed sperm viability and function. The study also aims to investigate how PRDX5 and PRDX6 protect sperm cells, focusing on their specific locations within the cel.

## Methods

### Chemicals and reagents

Protein-free BioXcell diluent (#016218) was obtained from IMV Technologies (L’Aigle, France), Sepharose CL-6B resin (#17,016,001) was purchased from Cytiva (Marlborough, MA, USA), CellMask Green (#C37608), the anti-CD9 antibody (#MA1-19,301), the anti-CD63 antibody (#MA5-28,419), the anti-CD81 antibody (#MA1-10,290) and the anti-TLR4 antibody (#76B357.1), the AP conjugated goat anti-mouse IgG (H + L) (#31,320) and Pierce 660 nm Protein Assay (#22,660) were obtained from Thermo Fisher Scientific (Waltham, MA, USA). The anti-PRDX5 antibody (#HPA037915), protease inhibitor cocktail (#P8340) urea (#51,456), thiourea (#T7875), 3-((3-cholamidopropyl)-dimethylammonio)-1-propanesulfonate (CHAPS) (#226,947), dithiotreitol (DTT) (#D0632), iodoacetamide (#I1149), sodium dodecyl sulfate (SDS) (#L3771), 2-mercaptoethanol (#M3148), Tween 20 (#P2287), the AP conjugated goat anti-rabbit IgG (#A3687), Nitro Blue Tetrazolium (NBT) (#N6876); N,N – dimethyloformamide (DMF) (#D4551), 5-Bromo-4-chloro-3-indolyl phosphate p-toludine salt (BCIP) (#B8503), merocyanine 540 (#M6390), and L carbonyl cyanide m-chlorophenylhydrazone (#857,815) were purchased from Sigma-Aldrich (St. Louis, MO, USA). Trypsin (#V511A) was obtained from Promega (Madison, USA). α-cyano-4-hydroxy-cinnamic acid (#28,166–41-8) was obtained from BrukerDaltonics (Bremen, Germany). Recombinant human peroxiredoxin 6 protein (#ab87631) was purchased from Abcam (Cambridge, UK). The anti-PRDX6 antibody (#MABN1797), DAF-FM DA (#251,520) and DEA NONOate (#D5431) were obtained from Merck Life Science (Darmstad, Germany). Yo-Pro™ -1 iodine P (#1304MP) was obtained from Invitrogen (Carlsbad, CA). Guava ® Mito Damage Kit was purchased from Luminex (Austin, TX, USA). APO-DIRECT™ Kit (#556381) was obtained from BD Biosciences Pharmingen (San Diego, CA, United States).

### Research material

Fresh semen was collected from eight sexually mature Holstein Friesian bulls from the Obrąb breeding farm, Poland, using an artificial vagina. The criteria for cryopreservation were as follows: a concentration atleast 1 × 10^9^ sperm cells/mL, sperm motility at least 70%. The semen was immediately diluted at a 1:2 ratio in protein-free Bioxcell diluent (IMV Technologies, L’Aigle, France). The diluted samples were transported to the laboratory at the Institute of Animal Reproduction, Polish Academy of Sciences (Olsztyn, Poland) at 4°C, arriving within 2 h of collection. Upon arrival, the samples were either stored at 4°C for further analysis or cryopreserved in straws. Prior to cryopreservation, the semen samples were diluted with Bioxcell diluent to a concentration of 50 million sperm cells per milliliter and kept at 4°C in a cooling chamber for 2 h to allow for equilibration. After equilibration, the samples were automatically filled into 0.25 mL straws (IVM Technologies, L’Aigle, France), positioned 4 cm above liquid nitrogen for 10 min, and then fully submerged and frozen in liquid nitrogen (− 196°C).

### Sperm concentration and viability measurement

Sperm concentration and viability from each bull were measured in three technical replicates using computer-aided fluorescent microscopy with the NucleoCounter SP-100 (Chemometec, Allerød, Denmark), following the protocol described by [16].

### Sperm motility measurement

Sperm motility in fresh semen was assessed immediately after collection and dilution tenfold with PBS. Motility in cryopreserved semen was evaluated after thawing the samples for 60 s at 37 °C. A 4 μL aliquot of semen was placed on a Leja glass slide (IMV Technologies Group, Nieuw-Vennep, The Netherlands) and mounted on a heated stage at 37 °C. Motility was analyzed using the computer-assisted sperm analysis system HT CASA II (CEROS II system, Hamilton-Thorne, USA). A minimum of 200 sperm cells per replicate were recorded, with measurements taken at a frame rate of 60 Hz (30 frames per second).

### Validation of anti-PRDX5 antibodies

The specificity of anti-PRDX5 antibodies was confirmed by mass spectrometry after 2D-PAGE and Western blotting. A total of 120 µg of sperm protein per sample was mixed with 25 mg/mL DTT, ampholytes, bromophenol blue, and rehydration buffer to 125 µL and loaded onto 7 cm IPG Immobiline DryStrips with a pH 3–10 gradient (GE Healthcare, Chicago, IL, USA). Strips were passively rehydrated for 12 h, followed by isoelectric focusing using the Ettan IPGphor [1] (GE Healthcare). After SDS-PAGE with 12% Mini-PROTEAN TGX Stain-Free Precast Gels (Bio-Rad, Hercules, CA, USA), stain-free gels were scanned using the ChemiDoc Touch Imaging System (Bio-Rad). Proteins were transferred to PVDF membranes, blocked with 5% non-fat dry milk in TBS-T, and incubated overnight with anti-PRDX5 antibodies. SameSpots software (Totallab, Newcastle, UK) was used to align protein spots. Gels were stained with Coomassie Brilliant Blue (CBB) G-250, and excised spots were digested with trypsin. Peptides were analyzed using a MALDI Autoflex Speed TOF/TOF mass spectrometer (Bruker Daltonics, Bremen, Germany) [1], confirming that the anti-PRDX5 antibody recognized peroxiredoxin 5 (PRDX5) from Bos taurus with 49% peptide sequence coverage.

### Validation of anti-PRDX6 antibodies

Anti-PRDX6 antibodies were validated using affinity purification since PRDX6 could not be identified via MALDI-TOF mass spectrometry due to nearby proteins. Anti-PRDX6 IgGs were coupled to an NHS-activated HP column (GE Healthcare). Sperm cells were separated from seminal plasma, washed, sonicated, and centrifuged. The sperm extract was applied to the column, and bound proteins were eluted with 0.5 M acetic acid and neutralized with 1 M Tris–HCl. The concentrated fractions underwent SDS-PAGE with 12% Mini-PROTEAN TGX Stain-Free Precast Gels (Bio-Rad), stained with Coomassie Brilliant Blue G-250. Protein bands were manually excised from the gels and analyzed by LC–MS, which confirmed the presence of peroxiredoxin 6.

### Fluorescent labeling of antibodies

To enable the antibodies used in the experiments to be analyzed by imaging flow cytometry, they were labeled with commercially available fluorescent dyes using the Conjugation Kit—Lightning-Link (Abcam, Cambridge, MA, USA), following the manufacturer’s instructions. The following labels were applied: anti-PRDX5 was conjugated with PE-Texas Red, anti-PRDX6 with PE-Cy7, anti-CD9 with PE-Cy5, and anti-CD63 with PE. The labeling procedure was as follows: 60 µl of antibodies at a concentration of 0.5 mg/ml were mixed with 6 µl of modifier reagent, and the mixture was transferred into a vial containing 60 µg of the respective fluorochrome. The reaction was incubated for 3 h at room temperature. After incubation, 6 µl of quencher reagent was added, followed by an additional 30-min incubation.

### Measurement of sperm quality parameters in fresh and cryopreserved semen

Flow cytometry analyses were performed using the Guava easyCyte™ System (EMD Millipore, Billerica, USA) containing two lasers operating at 488 nm (blue) and 642 nm (red) with filters presented in Table 1S. The guava InCyte™ module was used for sample acquisition and data analysis. The cell concentration was adjusted to be in the range of 200–300 cells/μL and total count of 5000 events were collected. The elimination of debris was based on cell size and the specific population of sperm cells was gated using forward scatter (FSC) and side scatter (SSC). Technical details can be found in the Supplementary Data.

### Fluidity analysis

1 × 10⁶ sperm cells diluted in PBS were incubated with 1.8 µM merocyanine 540, a probe for phospholipid scrambling (Sigma-Aldrich, product no. 323756), and 26 nM Yo-Pro™-1 iodide 491/509, a membrane-impermeable nucleic acid stain (Invitrogen, product no. Y3603) at 37 °C for 15 min. A positive control was performed by incubating sperm cells with 3% H₂O₂ for 1 h at 37 °C.

### Mitochondrial Membrane Potential (MMP)

MMP was measured using the Guava® Mito Damage Kit (Luminex, Austin, TX, USA, product no. FCCH100106) according to the manufacturer’s protocol. Semen was diluted in 1X Assay Buffer to a concentration of 1 × 10⁶ sperm/mL, and 100 μL of semen suspension was mixed with 100 μL of Mito Damage Working Solution containing MitoSense Red Dye, then incubated at 37 °C for 15 min. A positive control was performed by incubating sperm in 50 μmol/L carbonyl cyanide m-chlorophenylhydrazone (Sigma-Aldrich, product no. C2759) for 15 min at 37 °C.

### DNA fragmentation

DNA fragmentation was assessed using the APO-DIRECT™ Kit (BD Pharmingen™, product no. 556381) with slight modifications. Sperm cells (30 × 10⁶) were suspended in 4% paraformaldehyde (PFA) and kept on ice for 10 min. Following centrifugation and removal of PFA, the sperm pellet was suspended in 0.1% Triton X in PBS and incubated at 4 °C for 10 min. After washing, the sperm cells were incubated in a staining solution containing FITC-dUTP to label DNA breaks for 60 min at 37 °C. A positive control for DNA cleavage was prepared by treating fixed and permeabilized sperm with DNase I (10 U/mL) for 10 min at room temperature.

### Intracellular Nitric Oxide (NO) measurement

Intracellular NO levels were measured by incubating 1 × 10⁶ sperm cells diluted in dPBS with 25 µM DAF-FM DA (Sigma-Aldrich, product no. 251520) for 1 h at 37 °C. A positive control was generated by treating sperm with 1 mM DEA NONOate (Sigma-Aldrich, product no. D184) for 60 min at 37 °C.

### Reactive Oxygen Species (ROS) status

The ROS status of bovine sperm was measured using the CellROX Green reagent (Molecular Probes, Eugene, OR, USA). Aliquots containing 20 × 10⁶ cells/mL from fresh and cryopreserved sperm samples were taken, and the CellROX Green reagent (6.25 µM) was added. The cells were stained for 30 min at 38 °C. As a negative control, 3 mM N-acetylcysteine was used, while the positive control was obtained by using 2.5 mM menadione.

### Total and surface levels of PRDX5 and PRDX6 combined with ROS analysis

Fresh or cryopreserved semen samples (100 µl) containing 50 million sperm per ml were centrifuged at 350 × g for 10 min. The pellets were washed with 1 ml DPBS, centrifuged again at 350 × g for 10 min, and resuspended in 100 µl DPBS. To assess total levels of PRDX5 and PRDX6, 2.5 µl of 1% Triton was added to the sample. For surface-level analysis, 2.5 µl of DPBS was added instead. The samples were incubated for 10 min at room temperature. Next, 1 µl of fluorescently labeled anti-PRDX5 and anti-PRDX6 antibodies, along with 1 µl of the ROS indicator CellROX Green, were added to each sample. The samples were incubated for 1 h at 37 °C. After incubation, the samples were centrifuged at 350 × g for 10 min, washed with 1 ml DPBS, centrifuged again, and resuspended in 100 µl DPBS.

**Fig. 1:**
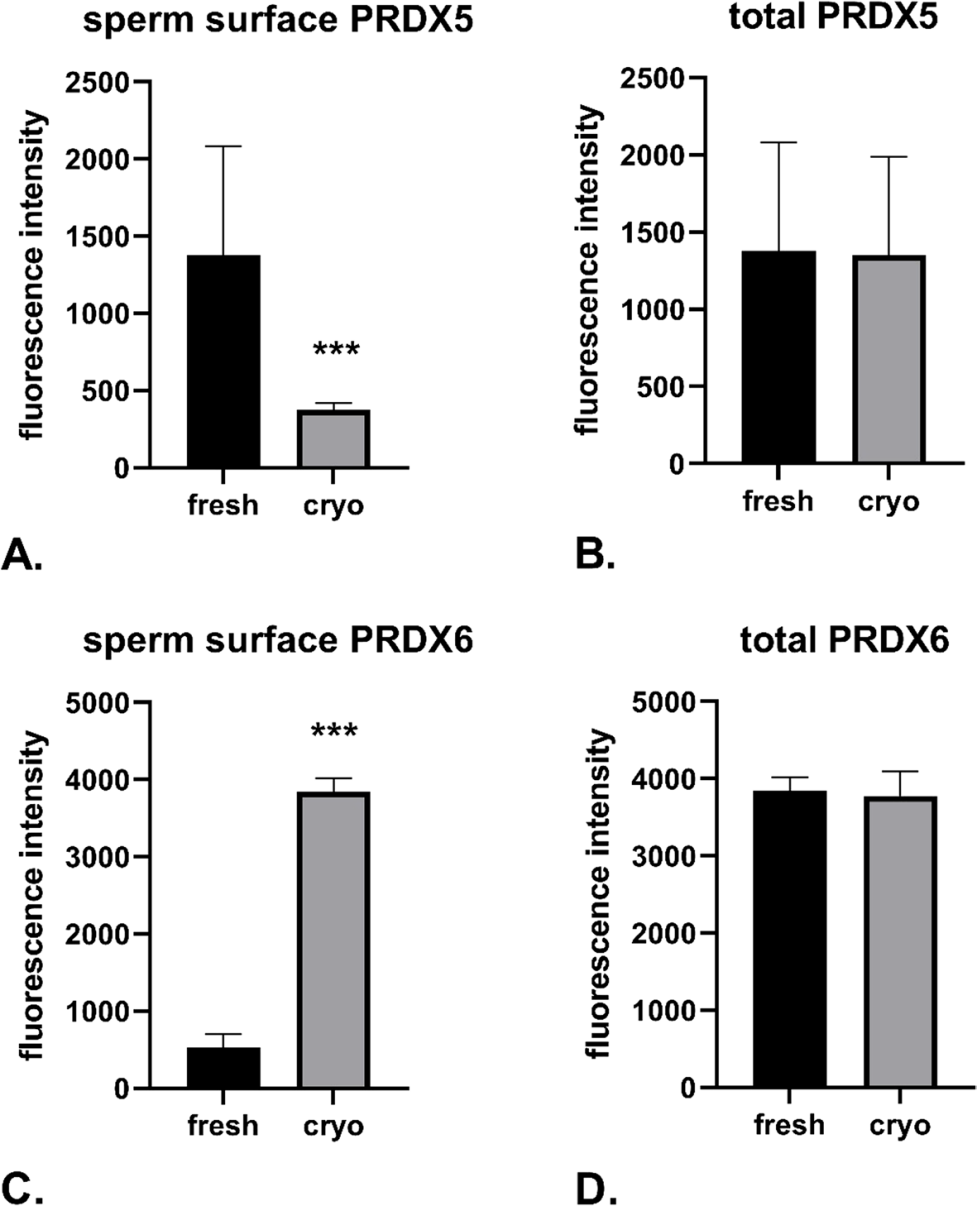
Presence of PRDX5 and PRDX6 on the sperm surface and within the entire sperm cell. Bar charts depict fluorescence intensity of fluorescently labeled anti-PRDX5 antibodies in non-permeabilized (**A**) and permeabilized (**B**) sperm samples, as well as anti-PRDX6 antibodies in non-permeabilized (**C**) and permeabilized (**D**) sperm samples. Data are presented as the mean ± SD (*n* = 8 sperm samples). ***p < 0.001

**Fig. 2:**
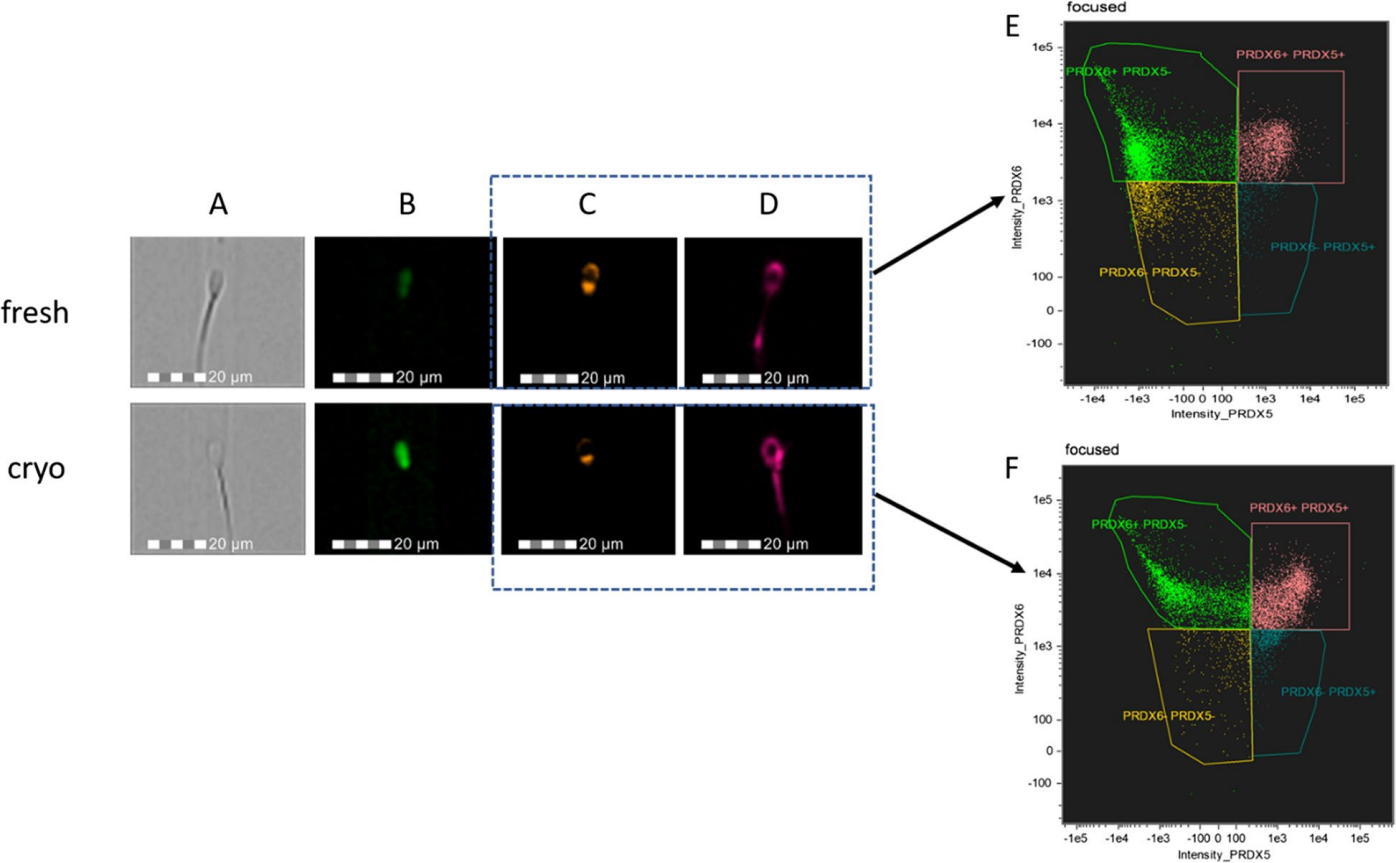
Imaging Flow Cytometry images of fresh and cryopreserved sperm, depicting the unstained sperm in Bright Field (**A**), stained for ROS + presence (**B**), labeled for PRDX5 on the sperm surface (**C**), and labeled for PRDX6 on the sperm surface (**D**). Corresponding Imaging Flow Cytometry plots illustrate sperm populations displaying the presence or absence of PRDX5 and PRDX6 in fresh (**E**) and cryopreserved (**F**) samples

The sperm samples were then analyzed using a FlowSight imaging flow cytometer (Cytek Biosciences, Fremont, CA, USA), with data acquisition performed using INSPIRE software (Cytek Biosciences, Fremont, CA, USA). Sperm cells were identified by plotting the brightfield area against the aspect ratio and were further verified using image galleries of the gated populations. CellROX Green, PE-Texas Red-conjugated anti-PRDX5, and PE-Cy7-conjugated anti-PRDX6 were excited by a 20 mW 488 nm laser. CellROX Green fluorescence was detected on Channel 2 (Ch02: 505–560 nm), PE-Texas Red-conjugated anti-PRDX5 on Channel 4 (Ch04: 595–660 nm), and PE-Cy7-conjugated anti-PRDX6 on Channel 6 (Ch06: 740–800 nm). For each sample, 10,000 events were collected. Data analysis was performed using IDEAS software (Cytek Biosciences, Fremont, CA, USA), with scatter plots used to evaluate the fluorescence of each fluorochrome, allowing simultaneous analysis of ROS levels, PRDX5, and PRDX6. In all cases, cell images were visually inspected to ensure the correct placement of gates (Figs. 1 and 2).

### Isolation and purification of EVs from seminal plasma

Fresh semen, as described earlier, was first centrifuged twice at 1500 g for 10 min at 4 °C. The supernatant was then diluted 1:2 in PBS and mixed thoroughly. The samples were subsequently centrifuged at 500 g for 10 min. The supernatant obtained from this step was then centrifuged again at 2000 g for 10 min. Finally, the supernatant underwent a high-speed centrifugation at 10,000 g for 15 min. The resulting supernatants were stored at -80 °C until EV isolation. All centrifugation steps were performed at 4 °C.

Next, the samples were concentrated to a volume of 500 µl using a centrifugal filter unit with a 10 kDa cutoff (Amicon Ultra-15 Centrifugal Filters, Merck Millipore) at 3000 g, again at 4 °C. The remaining flow-through after this step was used as exosome-depleted plasma.

For size-exclusion chromatography (SEC), Econo-Pac Chromatography columns (Bio-Rad) were packed with 15 ml of Sepharose CL-6B resin (Cytiva) and left for 3 h to allow the beads to settle and separate from the 20% ethanol. The columns were pre-washed twice with 15 ml of Milli-Q water, followed by 15 ml of PBS. A 500 µl concentrated sample was then added to the top of the column and allowed to pass through the filter on top of the Sepharose layer. Afterward, 10 ml of PBS was applied to the column. The SEC fractions were collected by eluting 21 fractions of 500 µl each into 1.5 ml microcentrifuge tubes. Each collected SEC fraction was analyzed for EV-specific characteristics.

### Extracellular vesicles identification

**Fig. 3:**
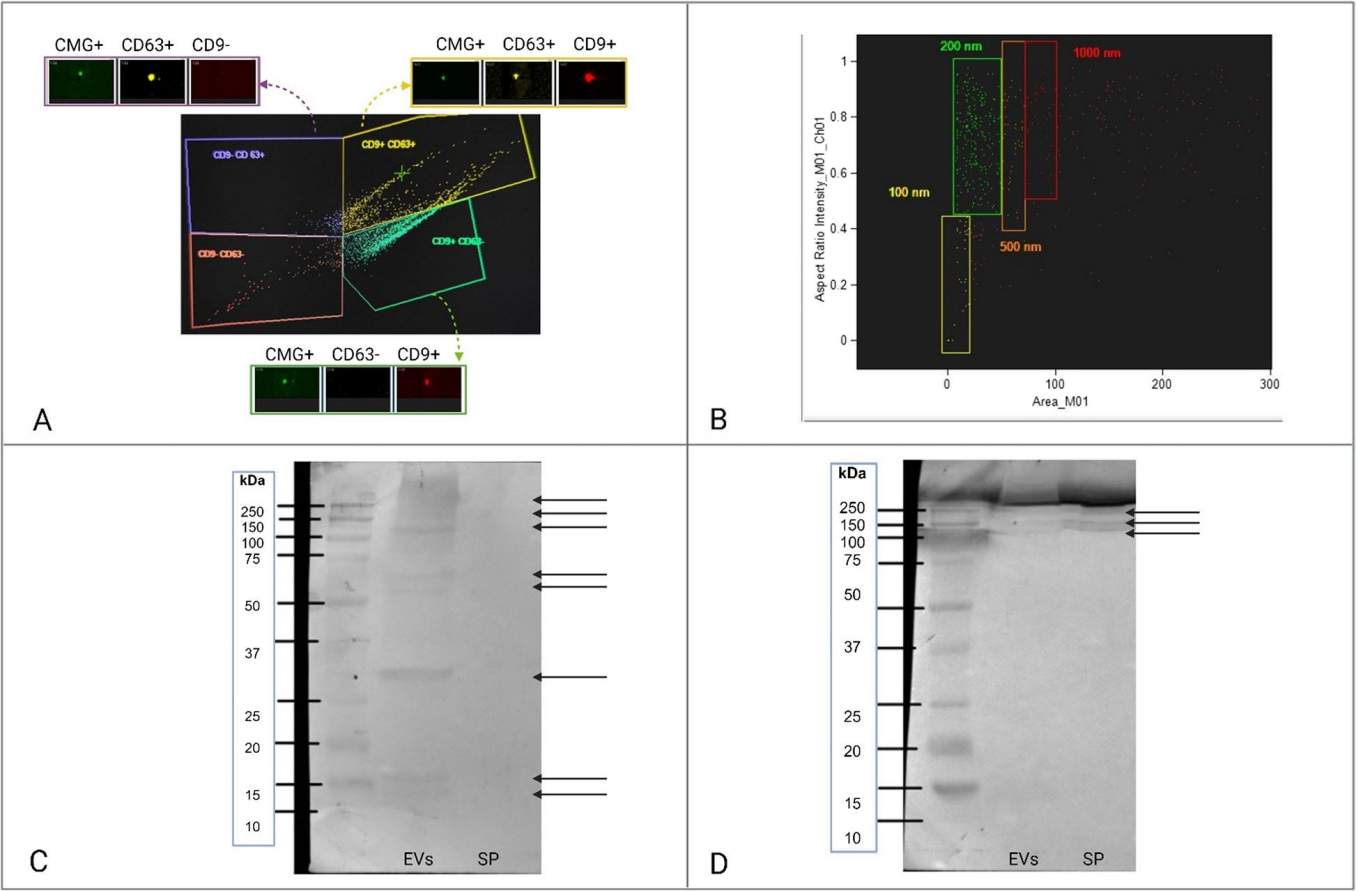
EV content, size, and PRDX5/PRDX6 expression in SEC fractions from bovine seminal plasma. In bovine seminal plasma SEC fractions 4–7, 72% of the detected particles were membranous (Cell Mask Green positive, CMG +). Of these, 61% expressed at least one EV-related surface marker (CD9 or CD63). The population marked in purple represents CMG + and CD63 + particles, the green population represents CMG + and CD9 + particles, and the yellow population represents CMG + , CD63 + , and CD9 + particles (**A**). EVs from SEC fractions 4–7 were sized between 100 and 200 nm (**B**). Western blot analysis of proteins separated by non-reducing SDS-PAGE, using anti-PRDX5 (**C**) and anti-PRDX6 (**D**) antibodies, demonstrated the presence of PRDX5 in the exosomal vesicle (EV) fraction, with no detectable signal in the exosome-depleted seminal plasma (SP). Arrows indicate 8 reactive bands for PRDX5 with wide MW range for PRDX5 in the EV fraction. In contrast, PRDX6 was detected in both the EV fraction (EVs) and the exosome-depleted seminal plasma (SP), with arrows indicating 3 reactive bands for PRDX6 with high MW for PRDX6, although the signal was weaker in both

The obtained SEC fractions were analyzed using a FlowSight imaging flow cytometer. To distinguish EVs from other particles, specific criteria were applied: particles had to be membrane-bound, indicated by Cell Mask Green positivity, and express at least one of the surface markers CD63 or CD9. EVs were incubated with Cell Mask Green, PE-CD63, and PE-Cy5-CD9 in a total volume of 20 μl (with 0.25 μl of each antibody and 2 μl of Cell Mask Green added to 17.5 μl EVs) for 45 min at 37 °C. Cell Mask Green, PE-CD63, and PE-Cy5-CD9 were excited using a 30 mW 488 nm laser. Fluorescence detection occurred as follows: Cell Mask Green on Channel 2 (Ch02: 505–560 nm), PE-CD63 on Channel 3 (Ch03: 560–595 nm), and PE-Cy5-CD9 on Channel 5 (Ch05: 642–740 nm). For each sample, 10,000 events were collected. Data analysis was performed using IDEAS software (Cytek Biosciences, Fremont, CA, USA), utilizing scatter plots to evaluate the fluorescence of Cell Mask Green, PE-CD63, and PE-Cy5-CD9. In all cases, images of the particles were visually inspected to ensure correct gating. The use of these three fluorescent markers enabled precise identification of the percentage of EVs among other particles and provided information on EV size (Fig. 3 A,B).

### Detection of PRDX5 and PRDX6 in EVs and exosome-depleted seminal plasma

Fractions 4–7, which were rich in EVs, were pooled and concentrated to 250 µl using an Amicon Ultra centrifugal filter unit with a 10 kDa cutoff (Merck Millipore). Both the EV samples and the flow-through samples (exosome-depleted seminal plasma) were heated for 5 min at 95 °C and separated using non-reducing SDS-PAGE on 12% Mini-PROTEAN TGX Stain-Free Precast Gels (Bio-Rad, Hercules, CA, USA). After electrophoresis, the proteins were transferred to PVDF membranes. The membranes were blocked in 5% non-fat dry milk dissolved in TBST and incubated overnight at 4 °C with primary antibodies: anti-PRDX5 (1:400) and anti-PRDX6 (1:400).

Following washing with TBST, the membranes were incubated with alkaline phosphatase-conjugated secondary antibodies (Thermo Fisher Scientific, Waltham, MA, USA) diluted 1:1000 for 1.5 h. The signal was developed using a mixture of NBT and BCIP with DMF (all purchased from Sigma-Aldrich, USA) dissolved in AP detection buffer in a dark room. Once the membranes dried, images were captured using the ChemiDoc Touch Imaging System (Bio-Rad) (Fig. 3 C,D).

### Detection of TLR4-PRDX complexes by immunoprecipitation

**Fig. 4:**
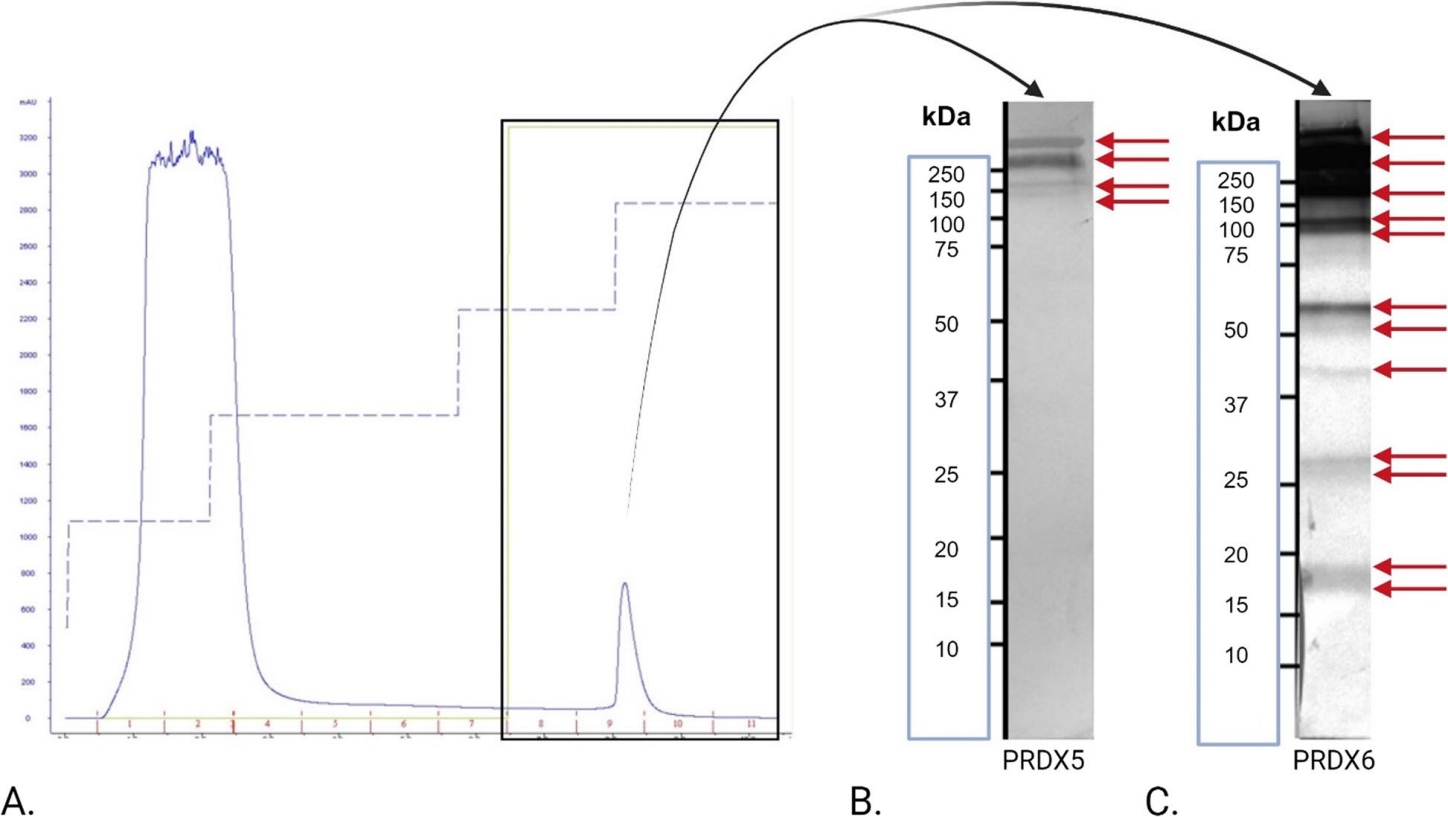
Identification of PRDX5 and PRDX6 complexes with TLR4 in sperm cells. Anti-TLR4 monoclonal antibodies were immobilized on a HiTrap protein A HP column (Cytiva) to capture TLR4-PRDX complexes from sperm lysates. The chromatogram highlights the fraction containing protein complexes associated with immobilized TLR4 (**A**). After elution and concentration, the immunoprecipitated proteins were analyzed by non-reducing SDS-PAGE, followed by Western blotting. Four distinct reactive bands corresponding to PRDX5 were detected above 150 kDa (**B**), while 12 reactive bands corresponding to PRDX6 were observed, ranging from 17 kDa to above 250 kDa (**C**). The reactive bands are indicated by red arrows. These results confirm the association of PRDX5 and PRDX6 with TLR4, indicating the formation of TLR4-PRDX complexes in sperm cells

To detect TLR4-PRDX complexes, anti-TLR4 monospecific IgGs were immobilized on a HiTrap Protein A HP column (Cytiva). Anti-TLR4 monoclonal antibodies (1.0 mg/mL, 100 µg) (Thermo Fisher Scientific, Waltham, MA, USA) were diluted in 20 mM sodium phosphate buffer (pH 7.0) and applied to the column. Sperm lysates, prepared as described earlier for native protein extraction, were also diluted in 20 mM sodium phosphate buffer (pH 7.0) and passed through the column with the immobilized antibodies. The antibody-antigen complexes were then eluted from the column using 0.1 M citric acid as the elution buffer. The eluted fractions were concentrated to 200 µl using Amicon Ultra centrifugal filters (3 kDa cutoff; Merck Millipore) (Fig. 4A). The immunoprecipitated proteins were subjected to SDS–polyacrylamide gel electrophoresis (SDS-PAGE), followed by immunoblotting to visualize peroxiredoxin 5 and 6 (Fig. 4B, C), following the Western blotting procedure in another section.

### Native, non-denaturing, and non-reducing PAGE

The isolation of native proteins from sperm was conducted following the method outlined by [17]. Fresh or thawed sperm cells were centrifuged at 800 × g for 10 min, washed twice with ice-cold PBS, and resuspended in PBS containing 1% protease inhibitor cocktail. The samples were sonicated on ice and centrifuged at 5000 × g for 15 min to isolate membrane proteins. The supernatant was collected after an additional sonication and centrifugation step. Proteins were precipitated with acetone (1:9 ratio) at -20°C for at least 2 h and resuspended in 0.15 M NaCl + 50 mM Tris buffer.

Protein concentration was measured using the Pierce 660 nm Protein Assay, and 30 µg of protein from each lysate was prepared in the appropriate modified Laemmli buffer for electrophoresis: Native PAGE: Buffer contained 31.25 mM Tris–HCl pH 6.8, 5% glycerol, and 0.002% bromophenol blue. Non-Denaturing PAGE: Buffer included 2.5% 2-mercaptoethanol without SDS. Non-Reducing SDS-PAGE: Buffer included 1.275% SDS without 2-mercaptoethanol. Proteins were resolved on 12% Mini-PROTEAN TGX Stain-Free Precast Gels and scanned using the ChemiDoc Touch Imaging System (Bio-Rad). Technical details can be found in the Supplementary Data.

### Standard SDS-PAGE

Denatured proteins were extracted from fresh or thawed sperm cells by centrifugation at 800 × g for 10 min at 4°C. Cells were lysed in a buffer containing 7 M urea, 2 M thiourea, 4% CHAPS, 1% protease inhibitor cocktail, and 50 mM DTT. The lysates were sonicated on ice and centrifuged at 4000 × g for 10 min. Proteins were purified using the Clean-Up Kit [17] and dissolved in rehydration buffer. For electrophoresis, 30 µg of protein was mixed with Laemmli buffer (31.25 mM Tris–HCl pH 6.8, 1.275% SDS, 5% glycerol, 2.5% 2-mercaptoethanol, and 0.002% bromophenol blue) and boiled for 5 min. Proteins were resolved on 12% Mini-PROTEAN TGX Stain-Free Precast Gels and visualized using the ChemiDoc Touch Imaging System (Bio-Rad). Technical details can be found in the Supplementary Data.

### Western blot analysis

Proteins were transferred from gels to PVDF membranes and scanned to obtain stain-free images. Membranes were blocked in 5% non-fat dry milk in TBST and incubated overnight at 4°C with primary antibodies (PRDX5 and PRDX6, 1:400). Detection was performed using alkaline phosphatase-conjugated secondary antibodies (1:5000) and visualized with Nitro Blue Tetrazolium (NBT) and BCIP solutions in AP detection buffer. Images were captured using the ChemiDoc Touch Imaging System, and protein quantification was normalized to total protein using stain-free technology. Technical details can be found in the Supplementary Data.

### Statistical analyses

The results are presented as the mean ± standard deviation (SD) (*n* = 8). Statistical analyses were conducted at significance levels of *p* < 0.05, 0.01, or 0.001 using GraphPad Prism software v6.02 (GraphPad Software Inc., San Diego, CA, USA). Data expressed as percentages were normalized using arcsine square root transformation. The normality of data distribution was assessed using the Shapiro–Wilk test, and homogeneity of variance was evaluated using Levene's test. For normally distributed data with equal variances, paired Student’s t-tests were used for comparisons. If the data did not meet the assumptions of normality, the non-parametric Wilcoxon signed-rank test was applied.

## Results

### Characterization of sperm quality parameters in fresh and cryopreserved samples

Cryopreservation significantly impacted several sperm quality parameters. The percentage of ROS-positive sperm increased over threefold after cryopreservation. The percentage of sperm showing DNA fragmentation increased more than threefold post-cryopreservation. The percentage of sperm with high mitochondrial potential decreased by 53% after cryopreservation. Furthermore, the percentage of NO-positive sperm increased sixfold following cryopreservation. Lastly, the percentage of sperm with high membrane fluidity increased fivefold post-cryopreservation (Table 1).

**Table 1:** Sperm quality parameters in fresh and cryopreserved semen: percentage of motile sperm (MOT), percentage of sperm exhibiting reactive oxygen species (ROS +), percentage of sperm with DNA fragmentation, percentage of sperm with decreased mitochondrial membrane potential (MMP), percentage of sperm positive for nitric oxide (NO +), and percentage of sperm with high membrane fluidity. Data are presented as the mean ± SD (*n* = 8 sperm samples). ***p < 0.001; **p < 0.01; *p < 0.05

Parameter	Fresh	Cryo
MOT [%]	80.4 ± 9.3	63.0 ± 6.7^***^
ROS + [%]	18.5 ± 3.8	45.4 ± 2.9^***^
DNA fragmentation [%]	21.6 ± 0.8	66.9 ± 2.5^***^
MMP [%]	39.6 ± 7.8	18.7 ± 4.8^**^
NO + [%]	0.04 ± 0.01	0.24 ± 0.01^*^
Membrane fluidity [%]	1.75 ± 0.2	8.6 ± 0.7^***^

### Impact of cryopreservation on surface and total levels of PRDX5 and PRDX6 in sperm

Cryopreservation affected the levels of PRDX5 and PRDX6 as measured by imaging flow cytometry. Surface levels of PRDX5 decreased post-cryopreservation (Fig. 1A), while total levels of PRDX5, including both intracellular and surface levels, remained unchanged (Fig. 1B). In contrast, surface levels of PRDX6 increased post-cryopreservation (Fig. 1C), with total levels of PRDX6, also remaining unchanged (Fig. 1D).

### Localization of PRDX5 and PRDX6 in fresh and cryopreserved bull semen

Imaging sperm cytometry facilitated simultaneous observation of anatomical structure (Fig. 2A), localization of ROS (Fig. 2B), localization of surface PRDX5 (Fig. 2C), and PRDX6 (Fig. 2D). In fresh semen, PRDX5 was primarily localized in the acrosomal cap and the post-acrosomal region. However, following cryopreservation, the signal diminished and was only detected in the post-acrosomal region (Fig. 2C). PRDX6 was found in the acrosomal cap, the post-acrosomal region, and the principal piece of the flagellum in fresh semen. Post-cryopreservation, the signal intensified and encompassed the entire surface of the cell membrane surrounding the head and mid-piece (Fig. 2D). Imaging flow cytometry plots revealed a decrease in the population of sperm exhibiting surface PRDX5 in fresh semen (Fig. 2E) and an increase in the population of sperm containing surface PRDX5 (Fig. 2F) post-cryopreservation. Quantitative comparison between surface PRDX5 and PRDX6 was shown in Fig, 1A and 1C.

### Presence of PRDX5 and PRDX6 in seminal plasma exosomal vesicles and exosome-depleted plasma

In addition to investigating PRDX5 and PRDX6 on the sperm surface and within the entire sperm cell, their presence in seminal plasma exosomal vesicles (EVs) and exosome-depleted seminal plasma (containing free-floating proteins) was also examined. SEC chromatography of previously filtered and concentrated seminal plasma yielded 21 distinct fractions. To differentiate EVs from other particles, SEC fractions were analyzed using an imaging flow cytometer based on the following criteria: membrane-bound properties (Cell Mask Green-positive) and the presence of at least one of two surface markers, CD63 and CD9 (Fig. 3A). The analysis revealed that SEC fractions 4–7 contained the highest percentage of EVs. Specifically, 72% of the detected particles were membrane-bound. Among these membrane-bound particles, 25% displayed both CD63 and CD9 markers, 45% had CD9 but not CD63, 5% had CD63 but not CD9, and 11% lacked both markers (Fig. 3A). The EVs in these fractions were found to be between 100 and 200 nm in size (Fig. 3B). Western blot analysis of the EV-positive mix from fractions 4–7 revealed the presence of PRDX5 in the EVs fraction of seminal plasma, with no detectable signal in the exosome-depleted plasma (Fig. 3C). In contrast, PRDX6 was detected in both the EV fraction and the exosome-depleted plasma, although the signal in EV fraction was weaker compared to PRDX5 (Fig. 3D).

### PRDX5 and PRDX6 form complexes with TLR4 in sperm cells

The immunoprecipitation analysis confirmed that both PRDX5 and PRDX6 form complexes with the surface protein TLR4 in sperm cells. Chromatographic analysis identified the specific fraction containing these protein complexes associated with immobilized TLR4 (Fig. 4A). Subsequent Western blot analysis of the immunoprecipitated proteins revealed four distinct bands corresponding to PRDX5, all above 150 kDa (Fig. 4B). In the case of PRDX6, twelve bands were observed, ranging from 17 kDa to above 250 kDa (Fig. 4C).

### Impact of cryopreservation on PRDX5 protein profiles

**Fig. 5:**
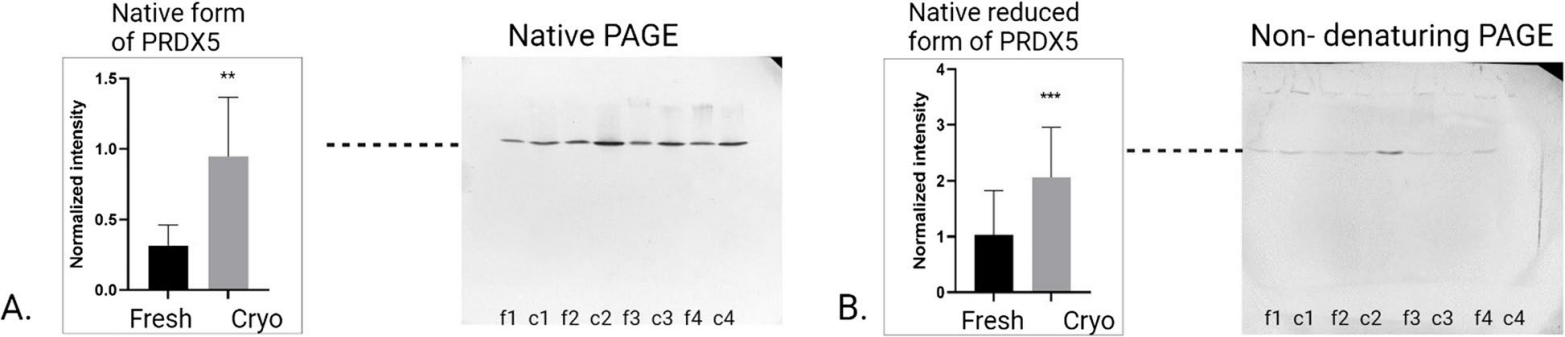
Graphs with corresponding representative Western blot analyses of PRDX5 under various electrophoretic conditions: (**A**) Native PAGE, showing a significant increase in the normalized intensity of the native band after cryopreservation (*p* < 0.01); (**B**) Non-denaturing PAGE, showing an increase in the normalized intensity of the native band after cryopreservation (*p* < 0.001). Lines f1-f4 correspond to fresh sperm samples, while c1-c4 correspond to cryopreserved sperm samples

**Fig. 6:**
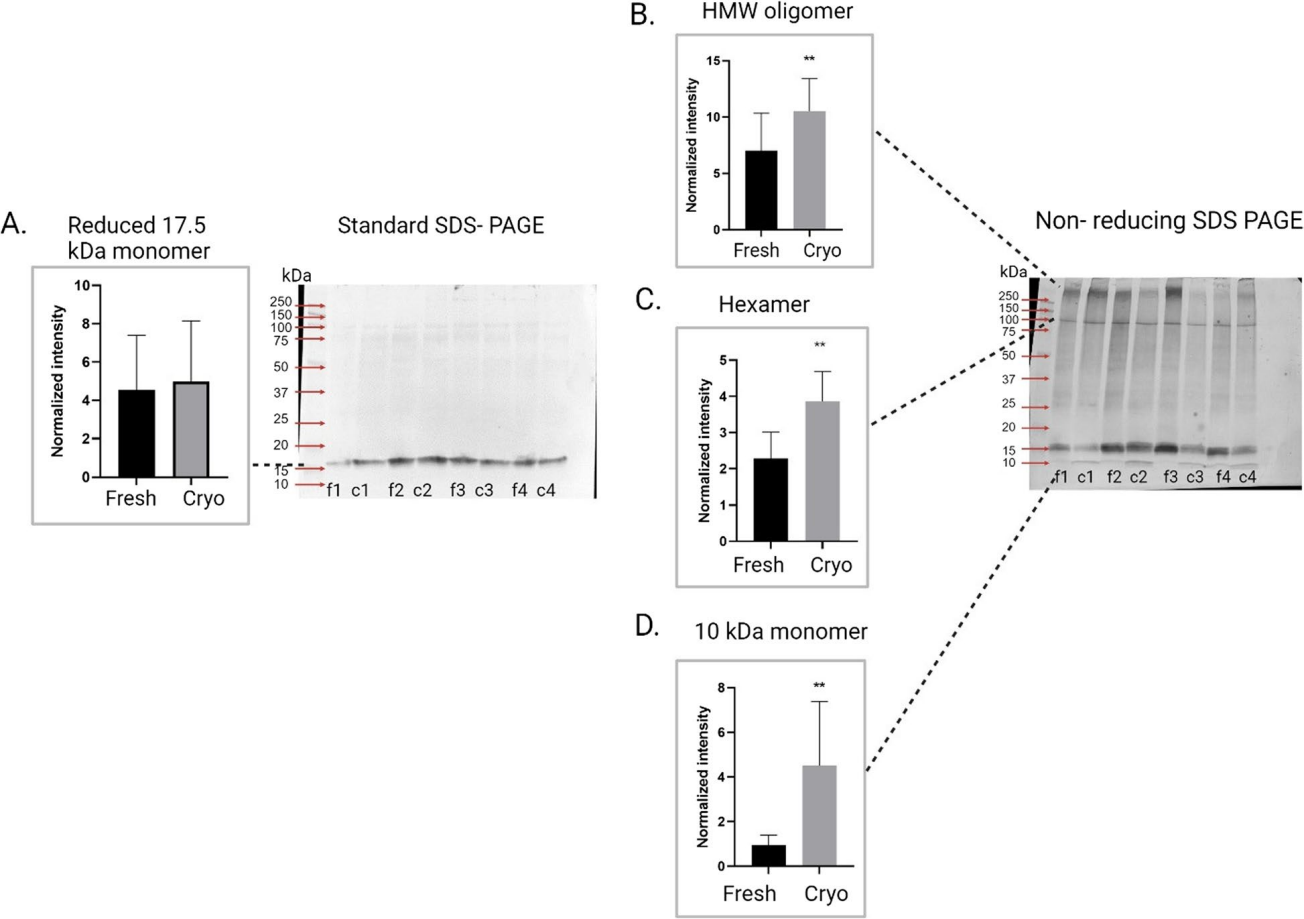
Graphs with corresponding representative Western blot analyses of PRDX5 under various electrophoretic conditions: Standard SDS-PAGE, with no changes in 17.5 kDa band intensity post-cryopreservation (**A**); Non-reducing SDS-PAGE, showing significant increases in the intensities of the > 250 kDa (**B**), 100 kDa (**C**) and 10 kDa (**D**) bands after cryopreservation (*p* < 0.05). Lines f1-f4 correspond to fresh sperm samples, while c1-c4 correspond to cryopreserved sperm samples

Native PAGE analysis revealed a significant increase in the intensity of a single band for PRDX5 following cryopreservation (*P* = 0.022) (Fig. 5A). Similarly, non-denaturing PAGE showed an increased intensity in native reduced form of PRDX5 (*p* < 0.001) (Fig. 5B). The band profiles observed in both Native PAGE and non-denaturing PAGE were consistent, with a single band detected by both methods. In contrast, standard SDS-PAGE showed no change in the intensity of the single 17.5 kDa band for PRDX5 after cryopreservation (Fig. 6A). Non-reducing SDS-PAGE analysis identified multiple bands for PRDX5, corresponding to molecular weights of 10, 15, 17.5, 100, and over 250 kDa (Fig. 6B, C, D). The intensities of the > 250 kDa (Fig. 6B),

100 kDa (Fig. 6C), and 10 kDa bands (Fig. 6D) significantly increased post-cryopreservation (*P* = 0.008, *P* = 0.0005, *P* = 0.005, respectively). The contrasting results between standard SDS-PAGE and non-reducing PAGE indicate that disulfide bonds are instrumental in the formation of PRDX5 oligomers, which are more prevalent under the oxidative stress conditions brought about by cryopreservation.

### Impact of cryopreservation on PRDX6 protein profiles

**Fig. 7:**
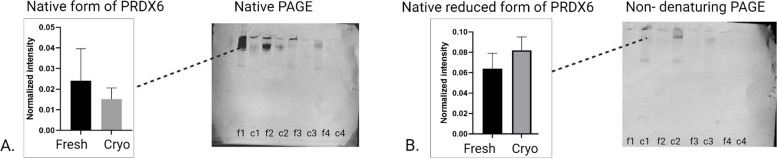
Graphs with corresponding representative Western blot analyses of PRDX6 under various electrophoretic conditions: (**A**) Native PAGE, showing no changes in the intensity of bands after cryopreservation; (**B**) Non-denaturing PAGE, showing no changes in the intensity of band post-cryopreservation; Lines f1-f4 correspond to fresh sperm samples, while c1-c4 correspond to cryopreserved sperm samples

**Fig. 8:**
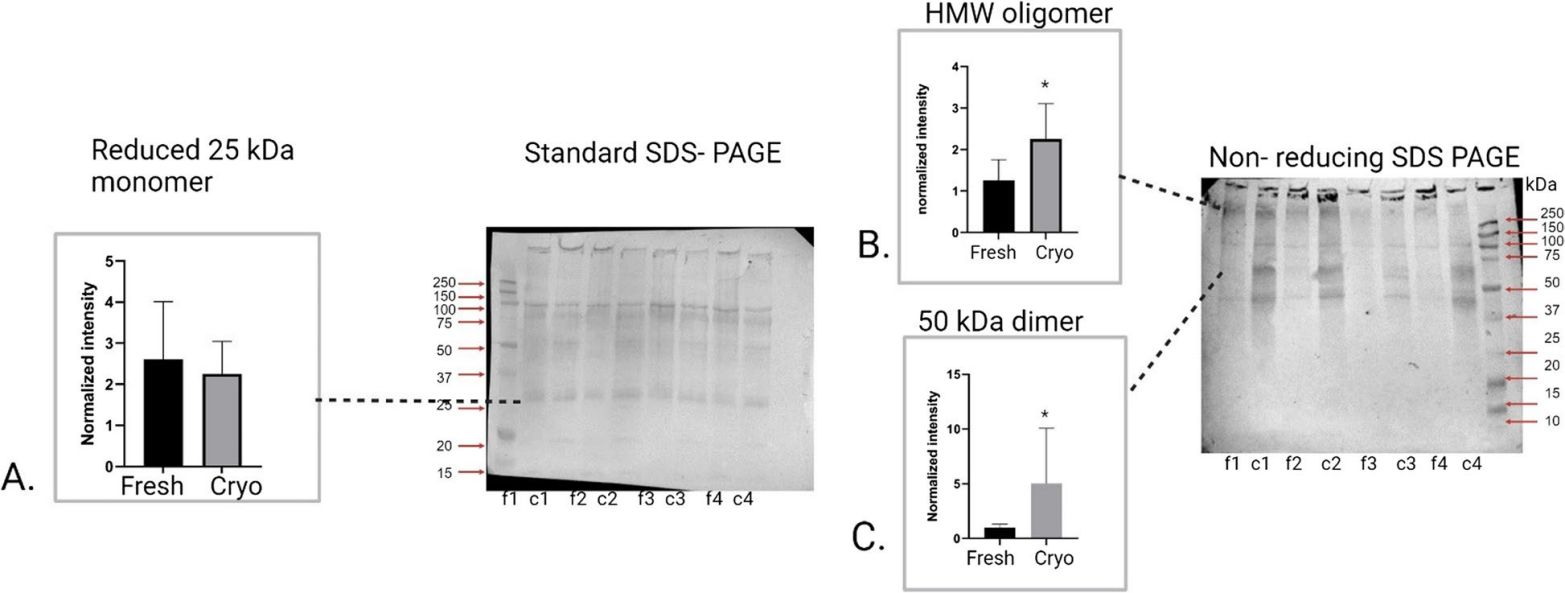
Graphs with corresponding representative Western blot analyses of PRDX6 under various electrophoretic conditions: (**A**) Standard SDS-PAGE, with no changes in the intensity of the single 25 kDa band after cryopreservation; Non-reducing SDS-PAGE, showing a significant increase in the intensity of > 250 kDa (**B**) and 50 kDa (**C**) bands after cryopreservation (*p* < 0.05). Lines f1-f4 correspond to fresh sperm samples, while c1-c4 correspond to cryopreserved sperm samples, while c1-c4 correspond to cryopreserved sperm samples

In the Native PAGE analysis, no significant differences were observed, although individual variability was evident among the samples (Fig. 7A). Non-denaturing PAGE showed a similar pattern of bands with unchanged intensity between treatments (Fig. 7B). Standard SDS-PAGE revealed no changes in the intensity of the single 25 kDa band for PRDX6 after cryopreservation (Fig. 8A). On the other hand, non-reducing SDS-PAGE analysis showed a significant increase in the intensity of bands above 250 kDa (Fig. 8B) and at 50 kDa (Fig. 8C) post-cryopreservation (*P* = 0.033; *P* = 0.044, respectively). The differences observed between standard SDS-PAGE and non-reducing SDS-PAGE suggest that disulfide bonds play a crucial role in the formation of PRDX6 oligomers, which become more prominent under oxidative stress conditions induced by cryopreservation.

## Discussion

### ROS accumulation, mitochondrial dysfunction and DNA Fragmentation as key outcomes of cryopreservation-induced Oxidative Stress

Cryopreservation, while essential for the long-term preservation of sperm, is known to induce significant oxidative stress, which has profound implications for sperm quality. Cryopreservation-induced oxidative stress was confirmed in the study by increased ROS + and NO + levels in sperm which were associated with decreased mitochondrial potential, increased DNA fragmentation, and elevated membrane fluidity. Elevated ROS levels are well-documented to cause oxidative damage to cellular structures, including lipids, proteins, and DNA, leading to compromised sperm function [18, 19]. Similarly, increased NO levels, which are often elevated in response to oxidative stress, have been linked to various detrimental effects on sperm, including alterations in mitochondrial function and membrane dynamics as shown in our previous study [7]. One of the key consequences of this oxidative stress is the significant decrease in mitochondrial membrane potential, a critical indicator of mitochondrial health and energy production. The mitochondria are not only the powerhouse of the cell but also a primary site for ROS generation, especially under stressful conditions like cryopreservation [20]. The loss of mitochondrial potential is closely associated with increased DNA fragmentation, which is a hallmark of oxidative damage to the genetic material of sperm [21]. This fragmentation can severely impair the fertilization potential and developmental competence of the sperm [22]. Furthermore, cryopreservation-induced oxidative stress may lead to changes in protein abundance and physical properties of the sperm membrane, notably increasing its fluidity [23]. Increased membrane fluidity shown in our study may be a result of lipid peroxidation, where ROS attack the polyunsaturated fatty acids in the membrane, causing disruptions in membrane integrity and function [24]. These changes in the membrane can affect key processes such as sperm motility and the ability to undergo the acrosome reaction, which is essential for fertilization.

### Differential translocation of PRDX5 and PRDX6 in response to cryopreservation-induced oxidative stress

Our results demonstrated that in response to the cryopreservation-induced oxidative environment, bovine PRDX5 and PRDX6 exhibited distinct patterns of translocation within sperm cells, with PRDX6 relocating to the cell membrane and PRDX5 moving intracellularly. Until now, the translocation of PRDX6 to the cell membrane in response to cryopreservation has only been documented in human sperm [2]. This phenomenon was also observed in somatic cells, where PRDX6 of lung epithelial cells similarly translocates to the membrane under oxidative stress conditions [25]. This translocation is thought to be a conserved protective strategy, driven by the phospholipase A2 (PLA2) activity of PRDX6, which plays a critical role in safeguarding cell membrane phospholipids from lipid peroxidation [26].

To our knowledge, our study is the first to introduce the novel observation that PRDX5, like PRDX6, undergoes translocation in sperm in response to cryopreservation. However, unlike PRDX6, the translocation of PRDX5 occurs in the opposite direction—from the surface of the sperm to its internal regions. This inward translocation of PRDX5 during cryopreservation likely plays a crucial role in protecting specific intracellular compartments from oxidative damage [7]. Key areas that may benefit from this protective mechanism include the mitochondrial sheath, which is vital for ATP production and sperm motility, and the nuclear region, where maintaining DNA integrity is essential for successful fertilization. Additionally, PRDX5 may target the acrosomal vesicle, safeguarding the enzymes necessary for oocyte penetration, as well as the plasma membrane, ensuring its fluidity and functionality. This redistribution of PRDX5 to internal compartments suggests a strategic antioxidant defense mechanism designed to preserve the viability and fertilization potential of sperm under the oxidative stress associated with cryopreservation.

### Oligomerization in peroxiredoxins: the role of disulfide bond oxidation in possible functional switching from peroxidase to chaperone activity

To our knowledge, this study is the first to demonstrate the formation of PRDX5 and PRDX6 oligomers in bovine sperm in response to oxidative stress induced by cryopreservation. Our research offers novel insights into the behavior of PRDX5 and PRDX6 during cryopreservation, particularly highlighting the role of disulfide bonds in oligomer formation. Standard SDS-PAGE analysis showed no changes in the intensity of monomer bands for PRDX5 and PRDX6 after cryopreservation, indicating the stability of these monomers. However, non-reducing SDS-PAGE revealed the appearance and significant intensification of bands larger than 250 kDa (HMW oligomer), 100 kDa (Hexamer), and 10 kDa (disulfide-linked monomer) for PRDX5, and bands larger than 250 kDa (HMW oligomer), and 50 kDa (dimer) for PRDX6. These findings, without corresponding changes in standard SDS-PAGE, strongly suggest that these oligomers are formed and stabilized by disulfide bonds in response to oxidative stress during cryopreservation.

Most studies on oligomer formation focus on other peroxiredoxin isoforms, such as PRDX1, PRDX2, PRDX4, or generally on the 2-Cys peroxiredoxin family. The formation of oligomers in PRDX1 and PRDX2 has been documented in human erythrocytes [27], PRDX3 in mitochondria of mammalian cells, and PRDX4 in the endoplasmic reticulum of human cells [28]. The formation of PRDX5 oligomers has been less explored, with the notable exception of a study by [9]. This study demonstrated that, under oxidative stress, cardiac PRDX5 shifts from a 17.5 kDa monomer to a smaller 10 kDa species, suggesting the formation of intramolecular disulfide bonds. This shift in molecular weight indicates that the oxidative environment induces a structural reconfiguration within PRDX5, leading to the creation of a disulfide-linked monomer, which is more compact and exhibits faster mobility on SDS-PAGE. These findings highlight a key mechanism by which PRDX5 adapts to oxidative stress, transitioning into a form that may help mitigate oxidative damage within the cell. Our research not only corroborates these observations but also expands on them by demonstrating that cryopreservation-induced oxidative stress leads to even more complex structural changes in PRDX5. In addition to the formation of the smaller 10 kDa species, we observed the development of larger oligomeric structures (> 250 kDa and 100 kDa) under non-reducing conditions. These larger oligomers likely result from both intra- and intermolecular disulfide bonding, indicating a more extensive aggregation process. This suggests that cryopreservation triggers a broader range of structural adaptations in PRDX5, potentially enhancing its protective functions through the stabilization of higher-order oligomers, which may act as molecular chaperones to prevent protein aggregation and maintain cellular integrity under stress conditions.

For PRDX6, we observed the emergence of oligomers with molecular weights around 50 kDa (dimer) and exceeding 250 kDa (hexamer). These results align with previous studies on rat brain, which have shown that PRDX6 oligomerization is closely linked to redox conditions and post-translational modifications [29]. Under hyperoxidation, PRDX6 can transition from a dimer to higher-order oligomers, a process confirmed by PAGE and dynamic light scattering experiments. This oligomerization not only alters the structural state of PRDX6 but also enhances its phospholipase A2 (iPLA2) activity, which plays a significant physiological role [29]. Furthermore, while the crystal structure of human PRDX6 is resolved as a dimer, solution studies on recombinant human PRDX6 suggest a dynamic equilibrium between monomers and dimers, influenced by the redox state [30]. This redox-dependent oligomerization is crucial for modulating PRDX6’s enzymatic functions, particularly under oxidative stress, and our results provide further evidence of its importance in the context of cryopreservation-induced stress.

Based on findings from somatic cells, we hypothesize that the oligomerization observed in bull sperm during cryopreservation leads to a functional shift toward chaperone activity. In somatic cells, oxidative stress triggers structural changes in PRDXs, like PRDX1, shifting their function from peroxidase to molecular chaperones, which are crucial for preventing protein aggregation under stress [31]. The formation of decamers and other higher-order structures, stabilized by disulfide bonds, is key to this chaperone activity. Disulfide bonds promote dimerization and the assembly of high-molecular-weight oligomers that exhibit chaperone functions. This process is tightly regulated by the cellular redox state, as shown by real-time observation techniques such as Förster resonance energy transfer (FRET). These studies highlight the importance of disulfide bond formation in switching PRDXs from peroxidase to chaperone activity [32]. We suggest that a similar mechanism occurs in bull sperm during cryopreservation, where oligomerization likely results in enhanced chaperone activity, helping to protect sperm from protein aggregation under oxidative stress.

Oligomerization-induced chaperone activity in PRDX5 and PRDX6 could be a critical factor in enhancing the resilience of sperm cells during cryopreservation. This chaperone function possibly helps maintain protein stability and prevents aggregation under the extreme oxidative stress associated with freezing and thawing processes. In our study, bull sperm—a species known for its high tolerance to cryopreservation—demonstrated the formation of these oligomers, suggesting that this mechanism may play a vital role in protecting sperm integrity and viability. The ability of PRDX5 and PRDX6 to form stable, disulfide-bonded oligomers may therefore be one of the key factors contributing to the success of bull sperm cryopreservation. However, further research is necessary to determine whether this protective mechanism is equally effective in the sperm of other species, where cryopreservation success rates may vary, and could potentially offer new insights into improving cryopreservation techniques across different species.

### Potential role of TLR4 in mediating the intracellular transport and functional adaptation of PRDX5 and PRDX6 under oxidative stress during cryopreservation

Our research reveals the formation of complexes between PRDX5, PRDX6, and Toll-like receptor 4 (TLR4), which may play a pivotal role in the intracellular transport of these peroxiredoxins, particularly under oxidative stress conditions induced by cryopreservation. Although no direct literature exists on the interaction of TLR4 with PRDX5 or PRDX6 in sperm cells, findings from studies on somatic cells strongly suggest that TLR4 could act as a key mediator in the intracellular trafficking and functional adaptation of PRDX5 and PRDX6. The disulfide form of PRDX5 has been shown to directly interact with TLR4, modulating inflammatory responses and facilitating its transport to areas of oxidative damage, such as in brain cells following injury. This interaction promotes both inflammatory and repair processes, underscoring the significance of TLR4 as a transport facilitator under stress conditions [13]. Similarly, in kidney cells, PRDX6 has been shown to utilize TLR4 to modulate inflammatory signaling while ensuring its proper localization to areas where its antioxidant function is most critical. This interaction significantly reduces oxidative stress and inflammation, improving mitochondrial function and cell viability, particularly in contexts of heightened oxidative stress, such as diabetic nephropathy [33]. These findings imply that in somatic cells TLR4 plays a dual role, not only in regulating immune responses but also in facilitating the intracellular trafficking of critical antioxidant proteins like PRDX5 and PRDX6 during oxidative stress.

The results of our study strongly suggest that a similar mechanism occurs in sperm cells, regulating inflammatory responses and aiding PRDX5 and PRDX6 relocation to sites of oxidative damage. Oxidative stress in fresh semen can be triggered by several factors, including infections, high levels of ROS from leukocytes, poor sperm handling, or environmental toxins. When oxidative stress occurs, the binding of TLR4 with PRDX5 or PRDX6 can initiate a protective response. Specifically, this interaction may lead to the translocation of PRDX proteins to key cellular compartments where they can mitigate oxidative damage, preserve mitochondrial function, and protect sperm DNA integrity. Understanding these interactions could pave the way for new strategies to enhance sperm resilience, particularly in assisted reproduction technologies.

### Potential role of exosomal vesicles in seminal plasma for PRDX5 and PRDX6 delivery and protection during cryopreservation

Our study has revealed the presence of PRDX5 and PRDX6 within EVs found in bovine seminal plasma, representing a significant advancement in understanding the mechanisms of sperm protection. To our knowledge, this is the first time that PRDX5 and PRDX6 have been identified in EVs derived from seminal plasma, suggesting a potentially novel mechanism for delivering critical antioxidant proteins to sperm cells. This discovery implies that EVs could serve as a transport system, ensuring that PRDX5 and PRDX6 reach sperm cells effectively, where they can provide essential protection against oxidative stress.

Previous studies on somatic cells have shown that exosomal pathways transport antioxidant enzymes under oxidative stress conditions. For instance, peroxiredoxins are secreted via exosomes in immune cells, playing a key role in modulating immune responses and protecting cells from oxidative damage [34]. There is limited information regarding the protein composition of sperm-derived EVs. However, it has been shown that PRDX-2, thioredoxin, and glutathione S-transferase are present in significantly higher quantities in EVs compared to whole ram seminal plasma [35]. This suggests that these vesicles play a crucial role in the exchange of proteins between sperm and their surrounding environment, which is vital for sperm function and fertility. The discovery of PRDX5 and PRDX6 within EVs from fresh bovine seminal plasma has important implications for understanding the mechanism of delivering these antioxidants precisely to the locations where they are most needed in sperm cells. This suggests that exosomal vesicles could play a crucial role in transporting and stabilizing antioxidant defenses within sperm, thereby enhancing the sperm’s ability to combat ROS and preserving critical structures such as membranes, mitochondria, and DNA. This delivery system may be vital for ensuring the optimal functioning and protection of sperm cells in response to environmental and oxidative stress. Future research should focus on whether the protective role of exosomal PRDX5 and PRDX6 extends to other species and reproductive systems, and how these findings could be leveraged to enhance fertility preservation techniques.

## Conclusions

This study sheds light on the responses of PRDX5 and PRDX6 in sperm cells under cryopreservation-induced oxidative stress. We observed distinct translocation patterns, with PRDX5 remaining intracellular, possibly to protect mitochondrial and genetic material, and PRDX6 relocating to the membrane, where it may play a role in protecting against oxidative damage. Furthermore, both PRDX5 and PRDX6 formed oligomers stabilized by disulfide bonds, suggesting a potential adaptive shift toward chaperone-like functions to support cellular structure under stress. In addition, we identified interactions between PRDX5, PRDX6, and TLR4, as well as their presence in extracellular vesicles, suggesting these proteins may contribute to a broader oxidative defense mechanism within the seminal plasma. This presence highlights potential routes for PRDX proteins in maintaining sperm integrity during cryopreservation.

In summary, our findings underscore a coordinated response of PRDX5 and PRDX6 to oxidative stress in cryopreserved sperm, expanding the understanding of antioxidant defenses in reproductive biology and suggesting new directions for improving sperm preservation techniques.

## Supplementary Information

Supplementary Material 1.

Supplementary Material 2.

Supplementary Material 3.

## Data Availability

No datasets were generated or analysed during the current study.

## Abbreviations

ROS: Reactive oxygen species
NO: Nitric oxide
PRDXs: Peroxiredoxins
iPLA2: Calcium-independent phospholipase A2
HMW: High-molecular-weight
TLR4: Toll-like receptor 4
LPS: Lipopolysacharide
EVs: Exosomal vesicles
SEC: Size-exclusion chromatography
PLA2: Phospholipase A2
FRET: Förster resonance energy transfer
MMP: Mitochondrial membranę potential
