# Supplementary material for: PRDX5 and PRDX6 translocation and oligomerization in bull sperm: a response to cryopreservation-induced oxidative stress
Source: Cell Commun Signal. 2025 Jan 9;23:15. doi: 10.1186/s12964-024-02015-9 (PMC11714857; doi:10.1186/s12964-024-02015-9)
Supplement: Supplementary file 1 — Supplementary Material 1. [file 12964_2024_2015_MOESM1_ESM.docx]

**Supplementary Data**

**Flow Cytometry**

Reagents and products used in flow cytometry analysis: Merocyanine 540 (Sigma-Aldrich, product no. 323756), Yo-Pro™-1 iodide 491/509 (Invitrogen, product no. Y3603), Guava® Mito Damage Kit (Luminex, product no. FCCH100106), APO-DIRECT™ Kit (BD Pharmingen™, product no. 556381), DAF-FM DA (Sigma-Aldrich, product no. 251520), and CellROX Green reagent (Molecular Probes). Additional reagents include carbonyl cyanide m-chlorophenylhydrazone (Sigma-Aldrich, product no. C2759), DEA NONOate (Sigma-Aldrich, product no. D184), and N-acetylcysteine.

For controls, fluidity analysis employed a positive control using 3% H₂O₂ incubated for 1 hour at 37°C. For mitochondrial membrane potential (MMP), a positive control was created with 50 μmol/L carbonyl cyanide m-chlorophenylhydrazone. DNA fragmentation used DNase I (10 U/mL) for 10 minutes at room temperature as a positive control. Intracellular nitric oxide (NO) levels were validated with a positive control of 1 mM DEA NONOate for 60 minutes at 37°C. Reactive oxygen species (ROS) measurements included a negative control with 3 mM N-acetylcysteine and a positive control with 2.5 mM menadione.

**Native, Non-Denaturing, and Non-Reducing PAGE**

Sperm cells were separated from the diluent by centrifugation at 800 × g for 10 minutes, followed by washing twice with phosphate-buffered saline (PBS) using an Eppendorf 5804R centrifuge. The cells were resuspended in 1% Triton X-100 PBS, sonicated five times for 10 seconds at 30% amplitude using a VCX-130 Ultrasonic Processor, and centrifuged at 5000 × g for 15 minutes to remove intracellular proteins. Membrane proteins were isolated by further sonication for 30 minutes (30 seconds on/off) in an Advantage-Lab™ Ultrasonic Water Bath and were precipitated by adding acetone in a 1:9 ratio. The samples were incubated at -20°C for 2 hours, centrifuged at 5000 × g for 1 hour, and resuspended in 0.15 M NaCl + 50 mM Tris buffer. Samples were stored at -80°C.

Electrophoresis buffers included 31.25 mM Tris-HCl pH 6.8, 5% glycerol, and 0.002% bromophenol blue for Native PAGE (no SDS or 2-mercaptoethanol). Non-Denaturing PAGE buffers additionally contained 2.5% 2-mercaptoethanol without SDS. For Non-Reducing SDS-PAGE, the buffer included 1.275% SDS without 2-mercaptoethanol.

**Standard SDS-PAGE**

Sperm cells were centrifuged at 800 × g for 10 minutes at 4°C, washed with PBS, and lysed in a buffer composed of 7 M urea, 2 M thiourea, 4% CHAPS, 1% protease inhibitor cocktail, and 50 mM dithiothreitol (DTT). Sonication was performed three times for 10 seconds at 30% amplitude on ice using a VCX-130 Ultrasonic Processor, followed by centrifugation at 4000 × g for 10 minutes. Lysates were purified with the Clean-Up Kit (GE Healthcare), and the final pellets were resuspended in rehydration buffer containing 7 M urea, 2 M thiourea, 2% CHAPS, and 50 mM DTT. Protein concentration was determined using the Pierce 660 nm Protein Assay, and absorbance was measured using a plate reader.

For electrophoresis, 30 µg of protein was suspended in Laemmli buffer containing 31.25 mM Tris-HCl (pH 6.8), 1.275% SDS, 5% glycerol, 2.5% 2-mercaptoethanol, and 0.002% bromophenol blue. Samples were boiled for 5 minutes and loaded onto 12% Mini-PROTEAN TGX Stain-Free Precast Gels. Gels were activated for 45 seconds and scanned using the ChemiDoc Touch Imaging System.

**Western Blot Analysis**

Proteins were transferred from gels to polyvinylidene fluoride (PVDF) membranes and scanned to generate stain-free images for preliminary normalization. Membranes were blocked in 5% non-fat dry milk dissolved in TBST (Tris-buffered saline with 0.1% Tween 20) for enhanced antibody binding. Primary antibodies used included peroxiredoxin 5 (PRDX5) and peroxiredoxin 6 (PRDX6) at a dilution of 1:400, while secondary antibodies conjugated to alkaline phosphatase (AP) were used at a dilution of 1:5000.

Detection was achieved with a mixture of Nitro Blue Tetrazolium (NBT) and 5-Bromo-4-chloro-3-indolyl phosphate (BCIP), dissolved in AP detection buffer (100 mM Tris-HCl, 100 mM NaCl, 5 mM MgCl₂, pH 9.5). Color development was stopped using 2 mM ethylenediaminetetraacetic acid tetrasodium salt dihydrate (EDTA). Once the membranes were dried, images were captured with the ChemiDoc Touch Imaging System, and band quantification was performed using Image Lab 5.0 software. Normalization was conducted using stain-free imaging technology, providing a reliable alternative to traditional housekeeping proteins.
