# Supplementary material for: PRDX5 and PRDX6 translocation and oligomerization in bull sperm: a response to cryopreservation-induced oxidative stress
Source: Cell Commun Signal. 2025 Jan 9;23:15. doi: 10.1186/s12964-024-02015-9 (PMC11714857; doi:10.1186/s12964-024-02015-9)
Supplement: Supplementary file 2 — Supplementary Material 2. [file 12964_2024_2015_MOESM2_ESM.docx]

Table 1. EasyCyte Lasers, Fluorescent Filters and Fluorochromes used in experiment

|  | Filter | Compatible fluorochromes used in the experiment |
| --- | --- | --- |
| **Laser 488 nm** |  |  |
| Green-Blue (GRN-B) | 525/30 | Yo-ProTM -1 iodine, FITC-dUTP, DAF-FM DA |
| Red-Blue (RED-B) | 695/50 | Merocyanine 540, PI |
| **Laser 642 nm** |  |  |
| Red-Red (RED-R) | 662/15 | MitoSense Red Dye |
