# Supplementary figures and images for: PRDX5 and PRDX6 translocation and oligomerization in bull sperm: a response to cryopreservation-induced oxidative stress
Source: Cell Commun Signal. 2025 Jan 9;23:15. doi: 10.1186/s12964-024-02015-9 (PMC11714857; doi:10.1186/s12964-024-02015-9)

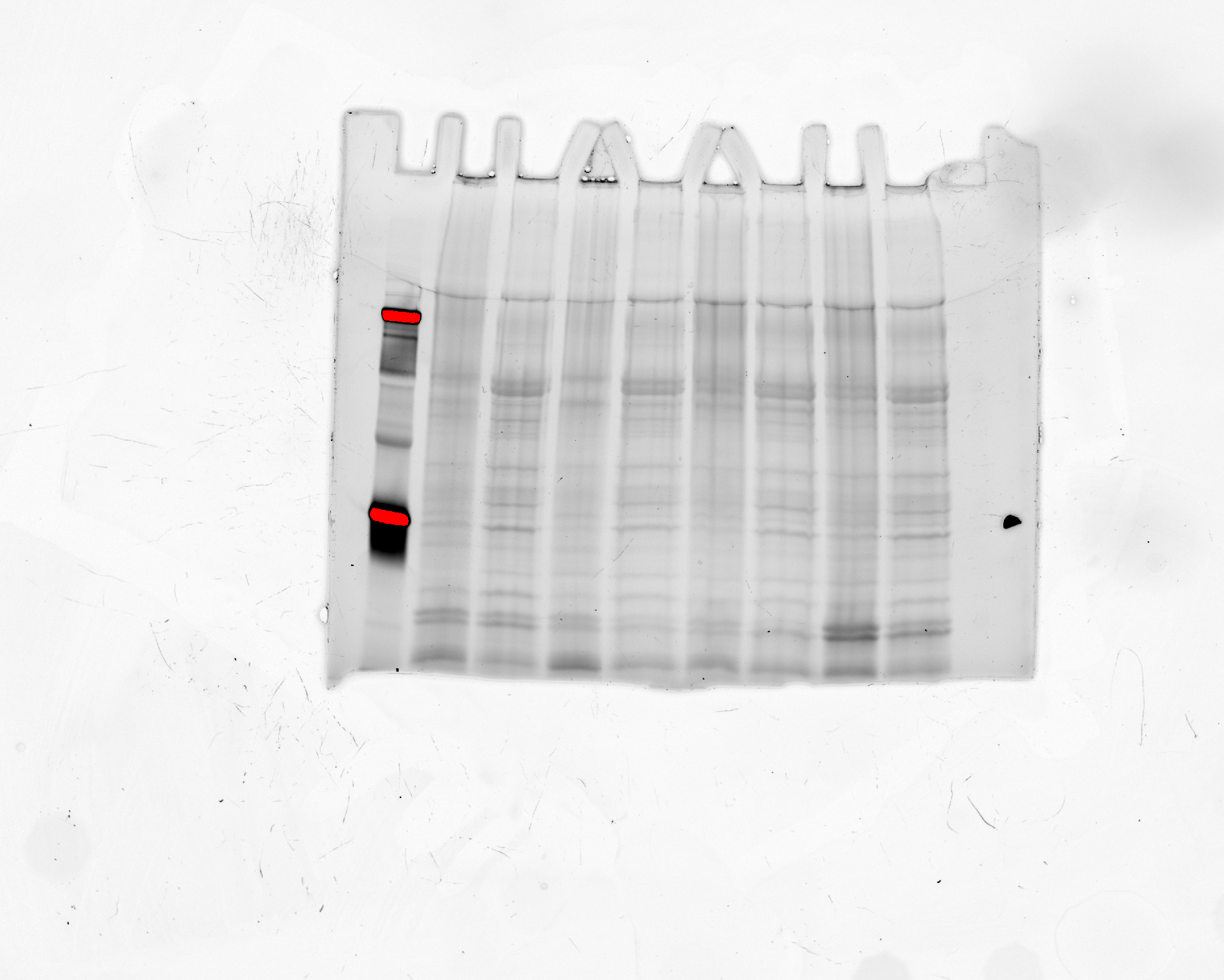

Supplement: Supplementary file 3 — Supplementary Material 3. [file 12964_2024_2015_MOESM3_ESM.zip › +SDS +red blot 1(Stain Free Gel) PRDX6.tif]

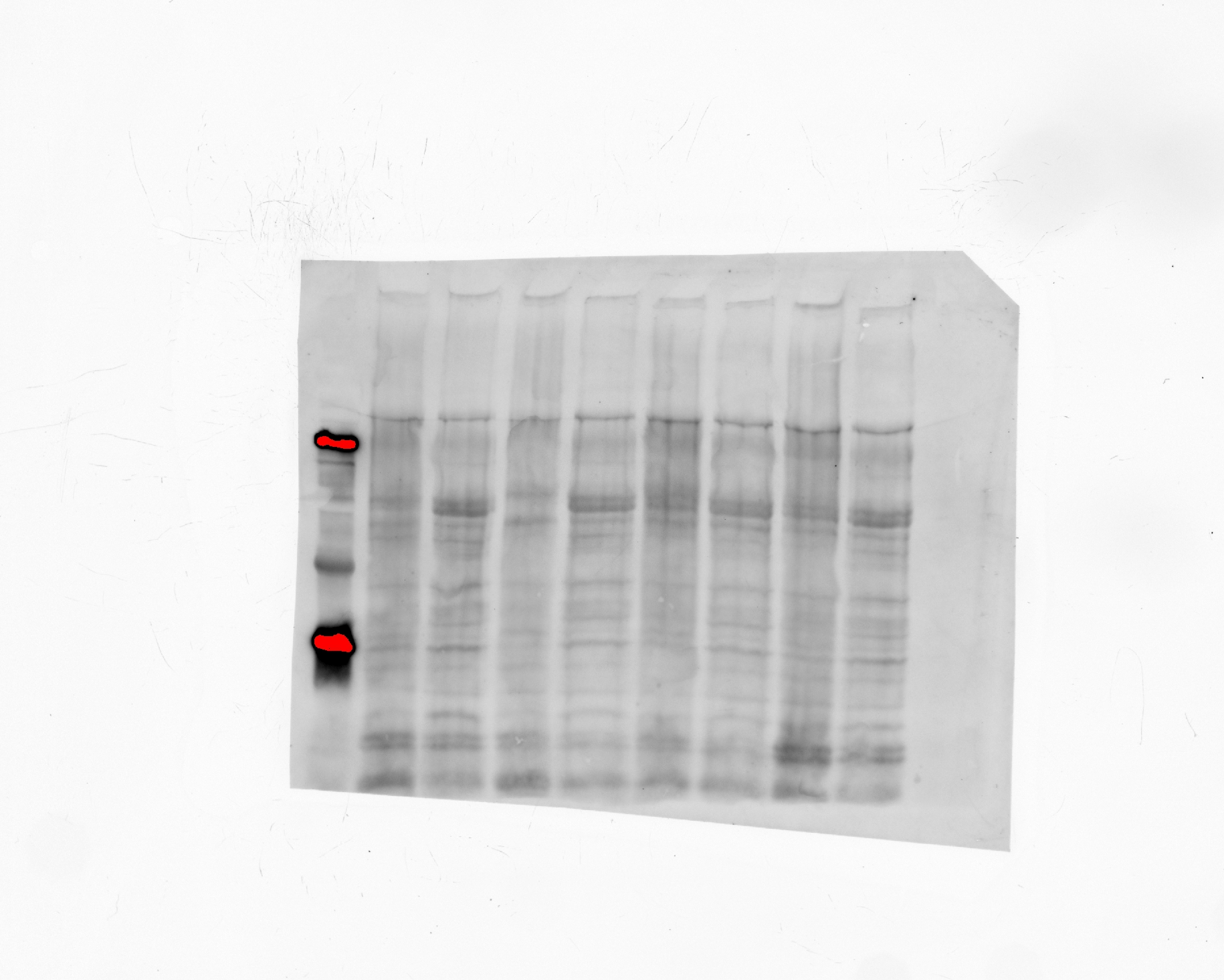

Supplement: Supplementary file 3 — Supplementary Material 3. [file 12964_2024_2015_MOESM3_ESM.zip › +SDS +redukt blot 1(Stain Free Blot) PRDX6.jpg]

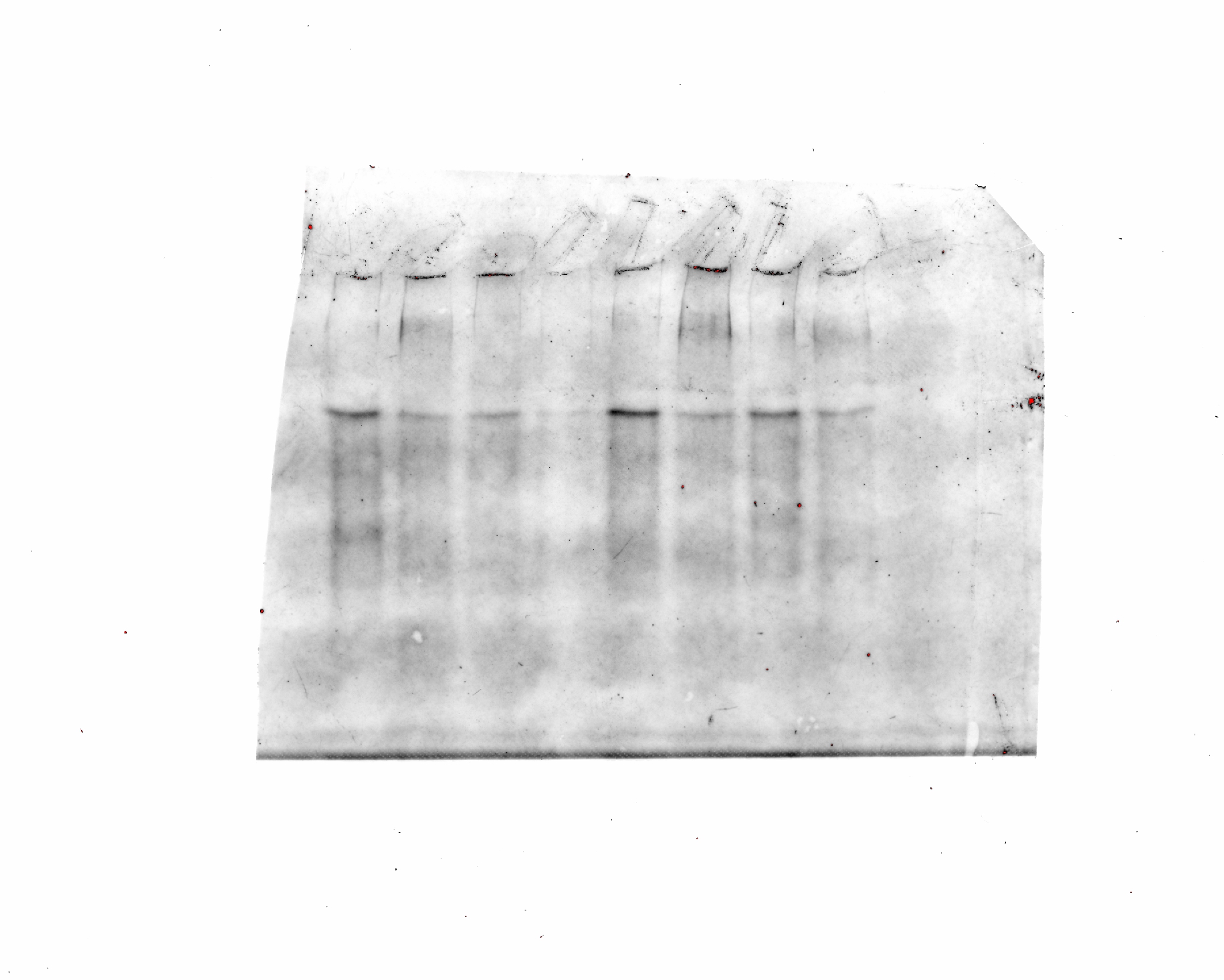

Supplement: Supplementary file 3 — Supplementary Material 3. [file 12964_2024_2015_MOESM3_ESM.zip › blot 1 (-SDS,+redukt) (Stain Free Blot) PRDX6.jpg]

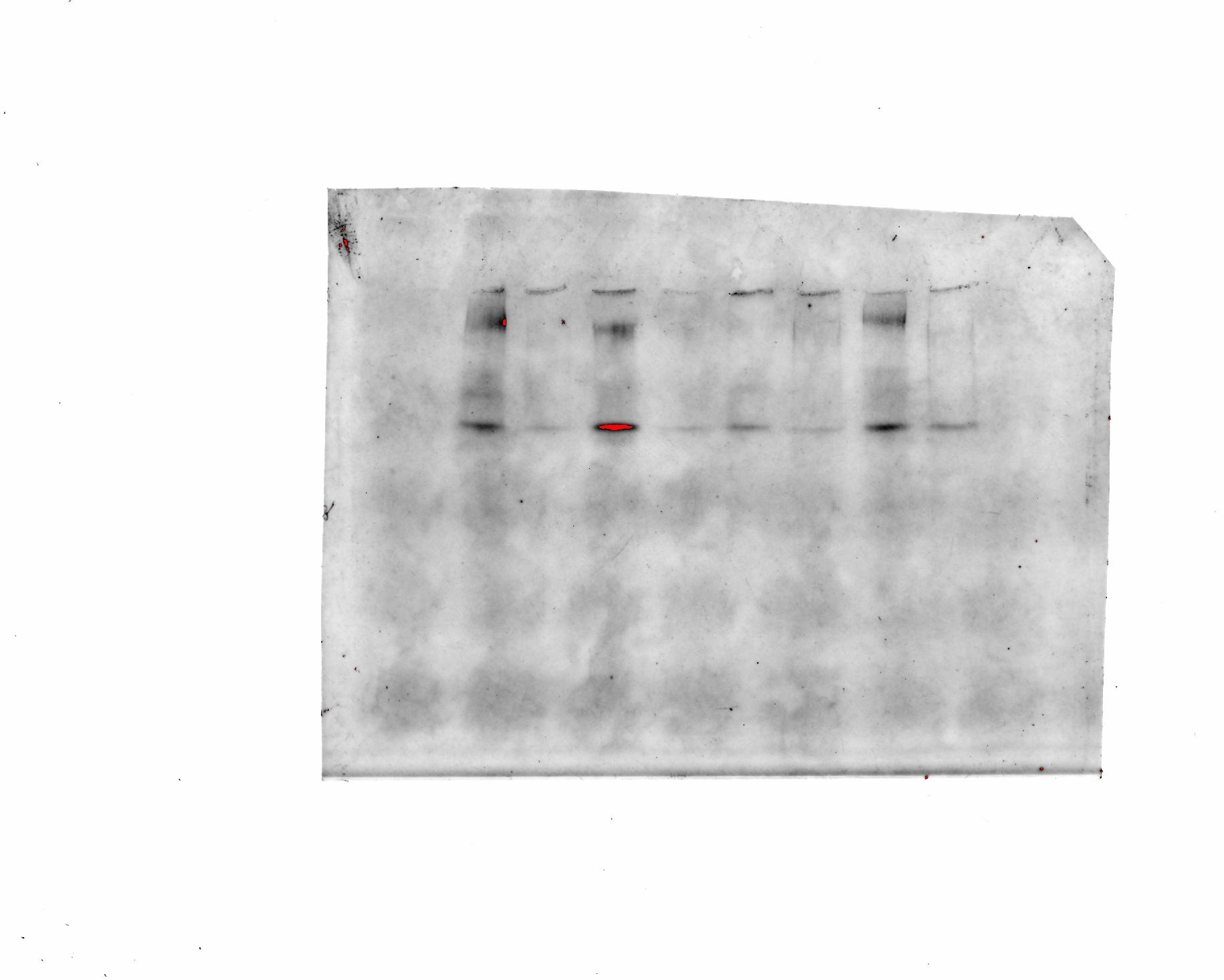

Supplement: Supplementary file 3 — Supplementary Material 3. [file 12964_2024_2015_MOESM3_ESM.zip › blot 1 (-SDS,-redukt)(Stain Free Blot) PRDX6.tif]

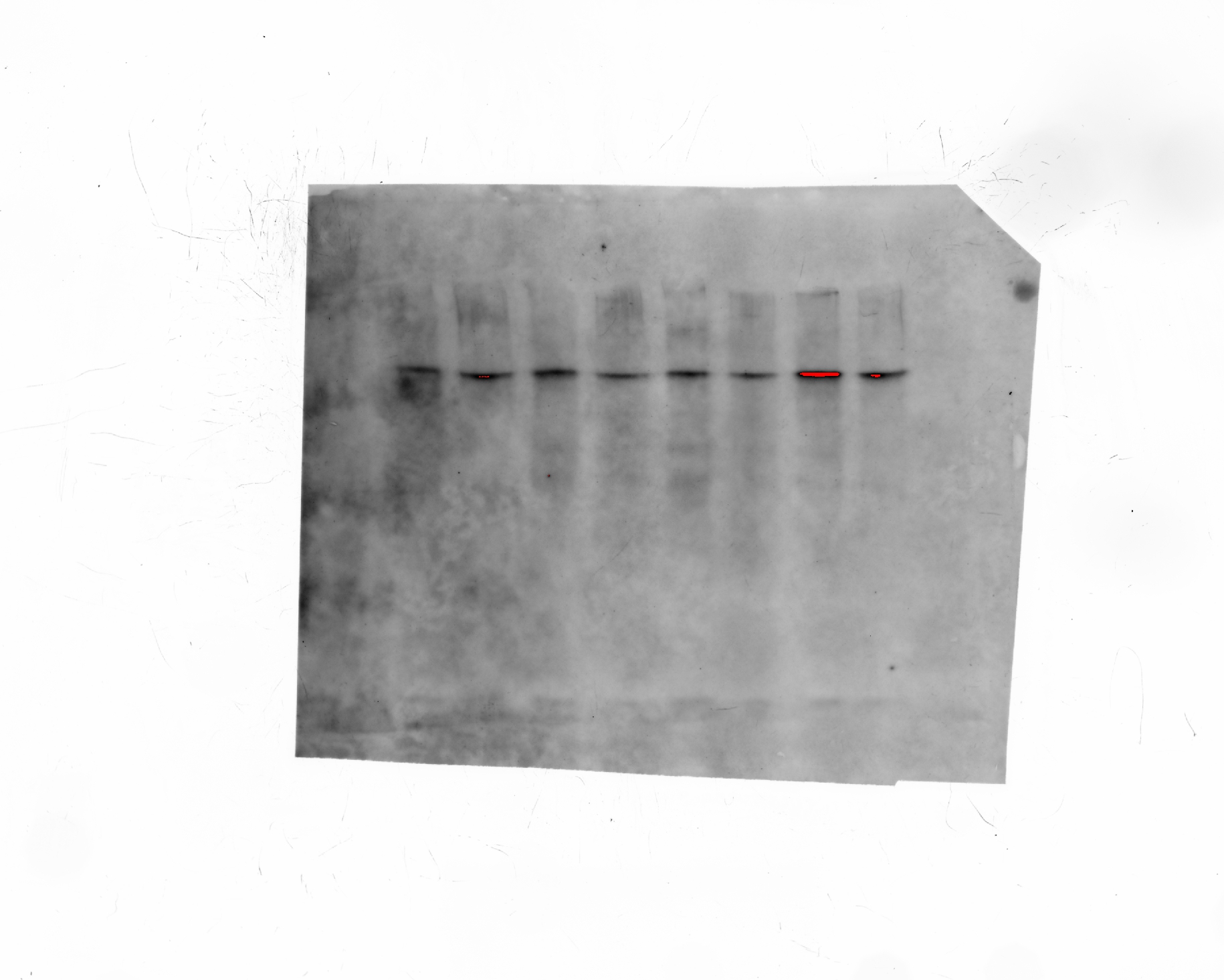

Supplement: Supplementary file 3 — Supplementary Material 3. [file 12964_2024_2015_MOESM3_ESM.zip › blot 1 (Stain Free Blot) PRDX5 native.jpg]

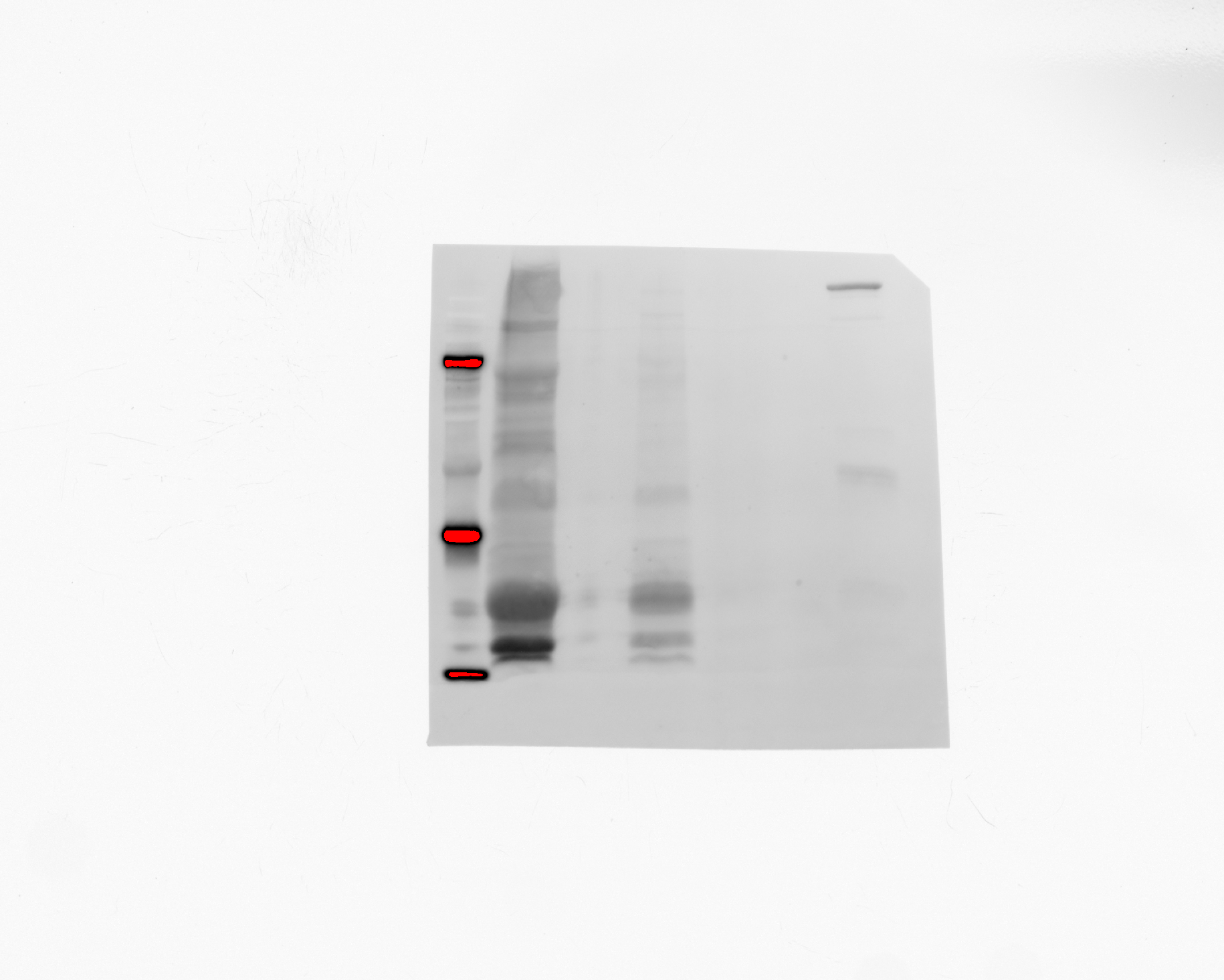

Supplement: Supplementary file 3 — Supplementary Material 3. [file 12964_2024_2015_MOESM3_ESM.zip › EVs+PRDX5 (Stain Free Blot).tif]

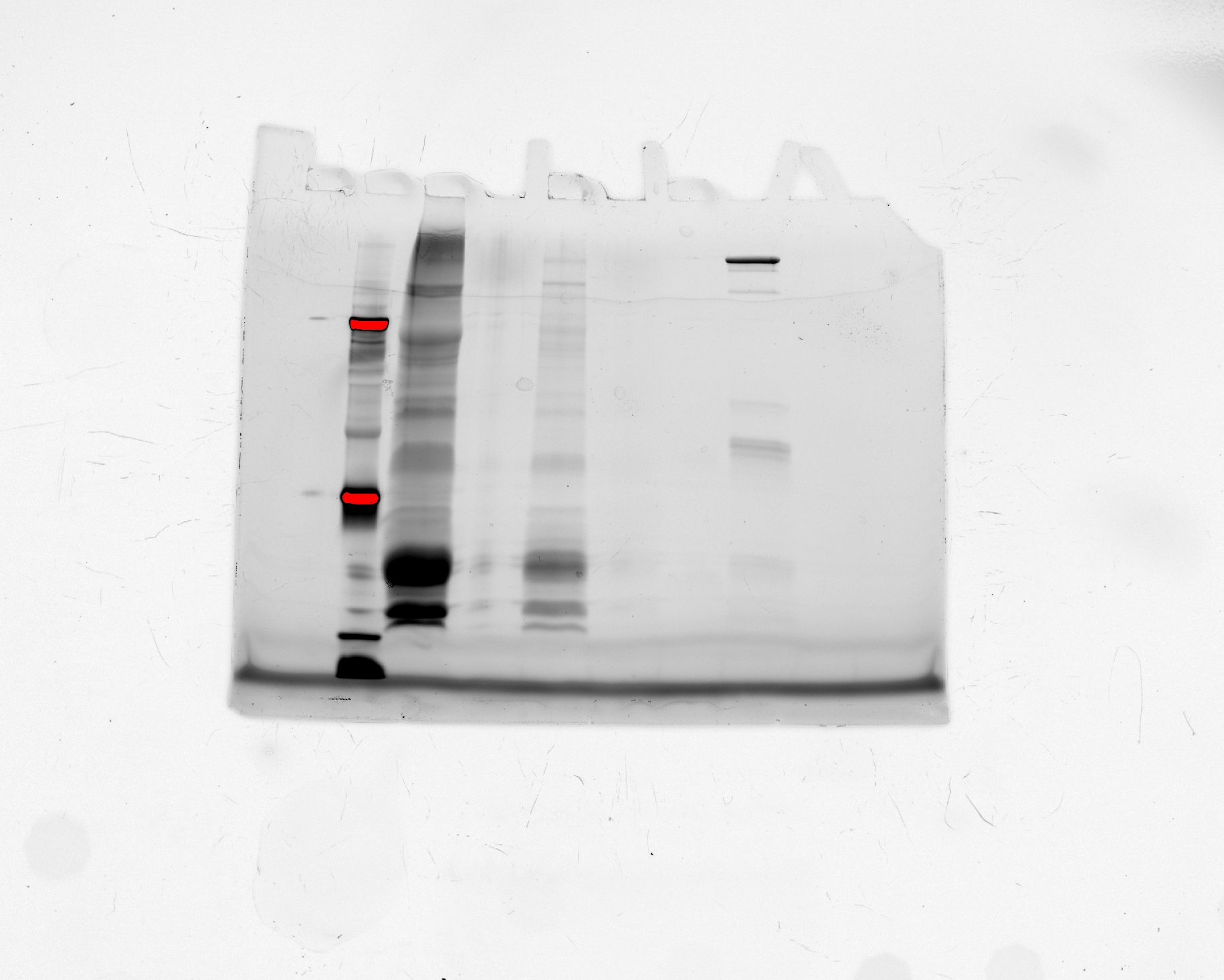

Supplement: Supplementary file 3 — Supplementary Material 3. [file 12964_2024_2015_MOESM3_ESM.zip › EVs+PRDX5 (stain Free Gel).jpg]

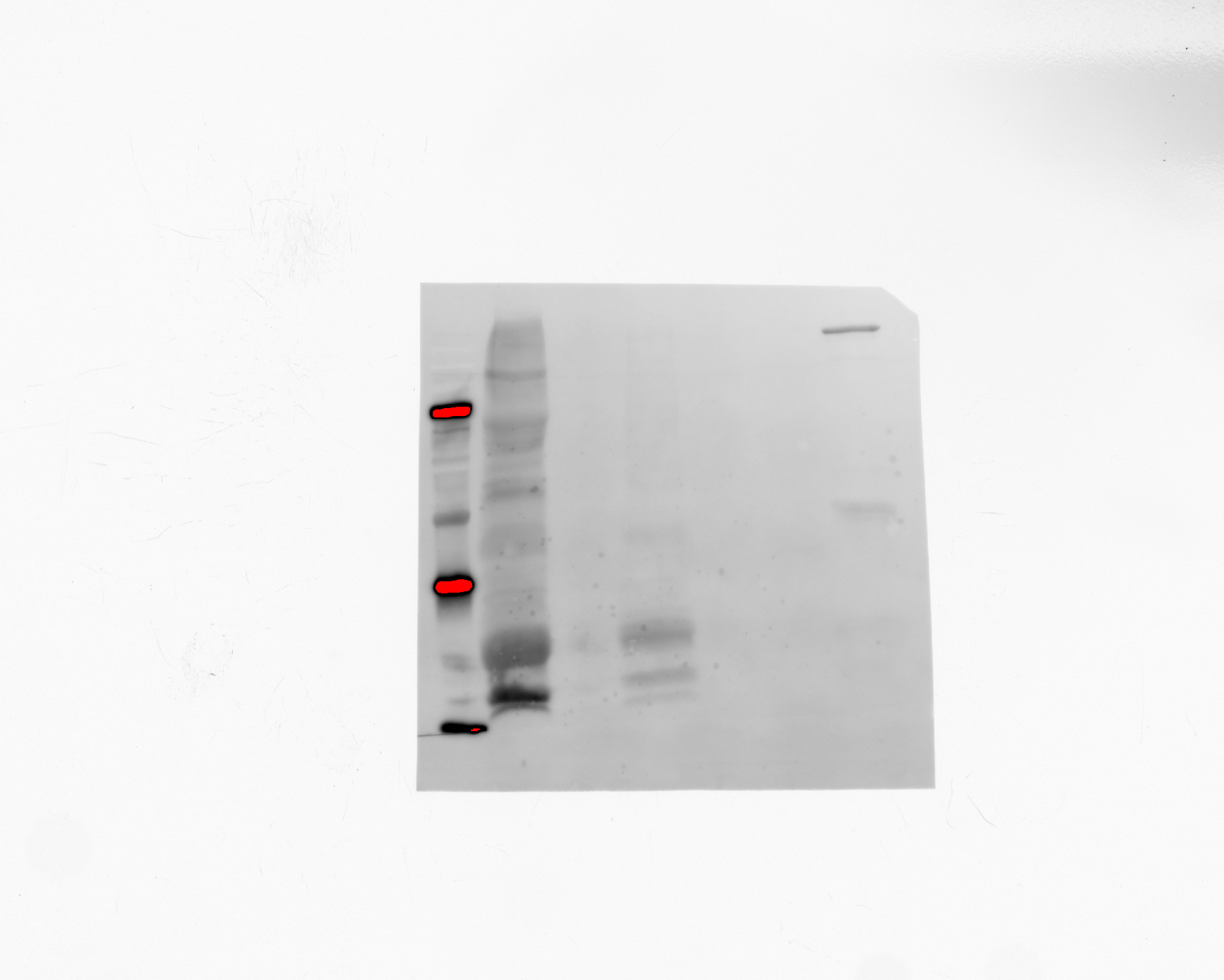

Supplement: Supplementary file 3 — Supplementary Material 3. [file 12964_2024_2015_MOESM3_ESM.zip › EVs+PRDX6 (Stain Free Blot).tif]

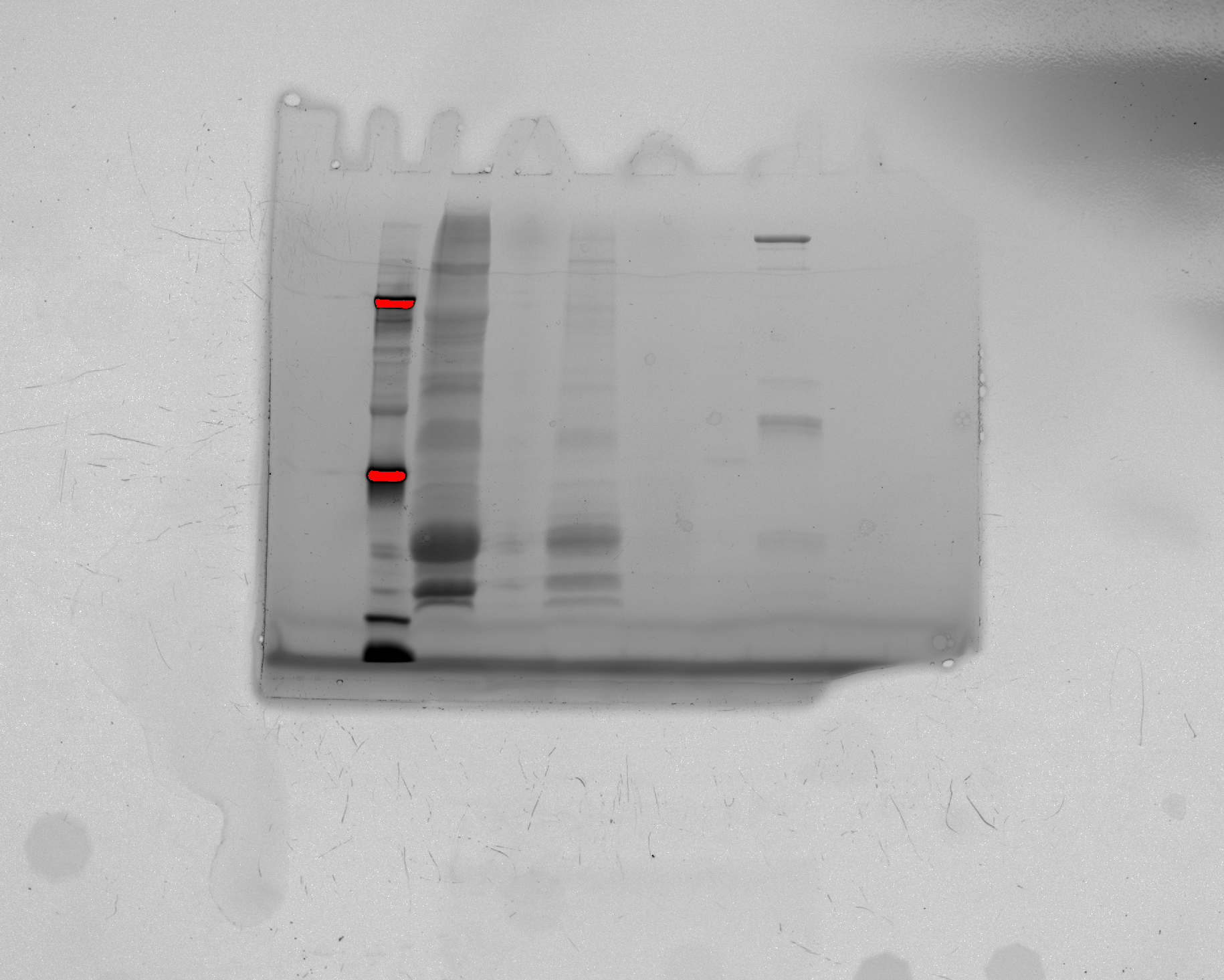

Supplement: Supplementary file 3 — Supplementary Material 3. [file 12964_2024_2015_MOESM3_ESM.zip › EVs+PRDX6 (Stain Free Gel).tif]

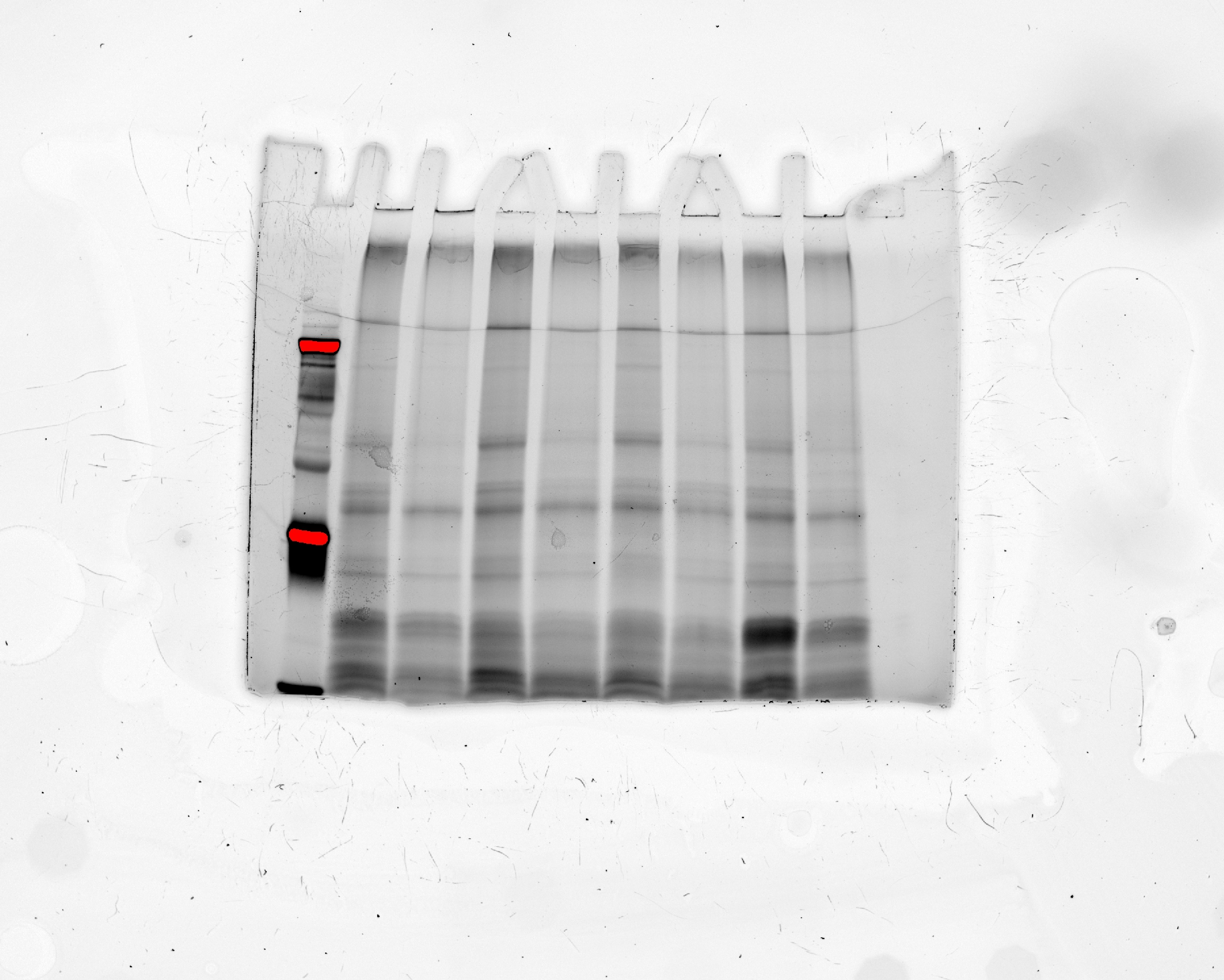

Supplement: Supplementary file 3 — Supplementary Material 3. [file 12964_2024_2015_MOESM3_ESM.zip › non reducing zel 1 (stain free Gel) PRDX5.jpg]

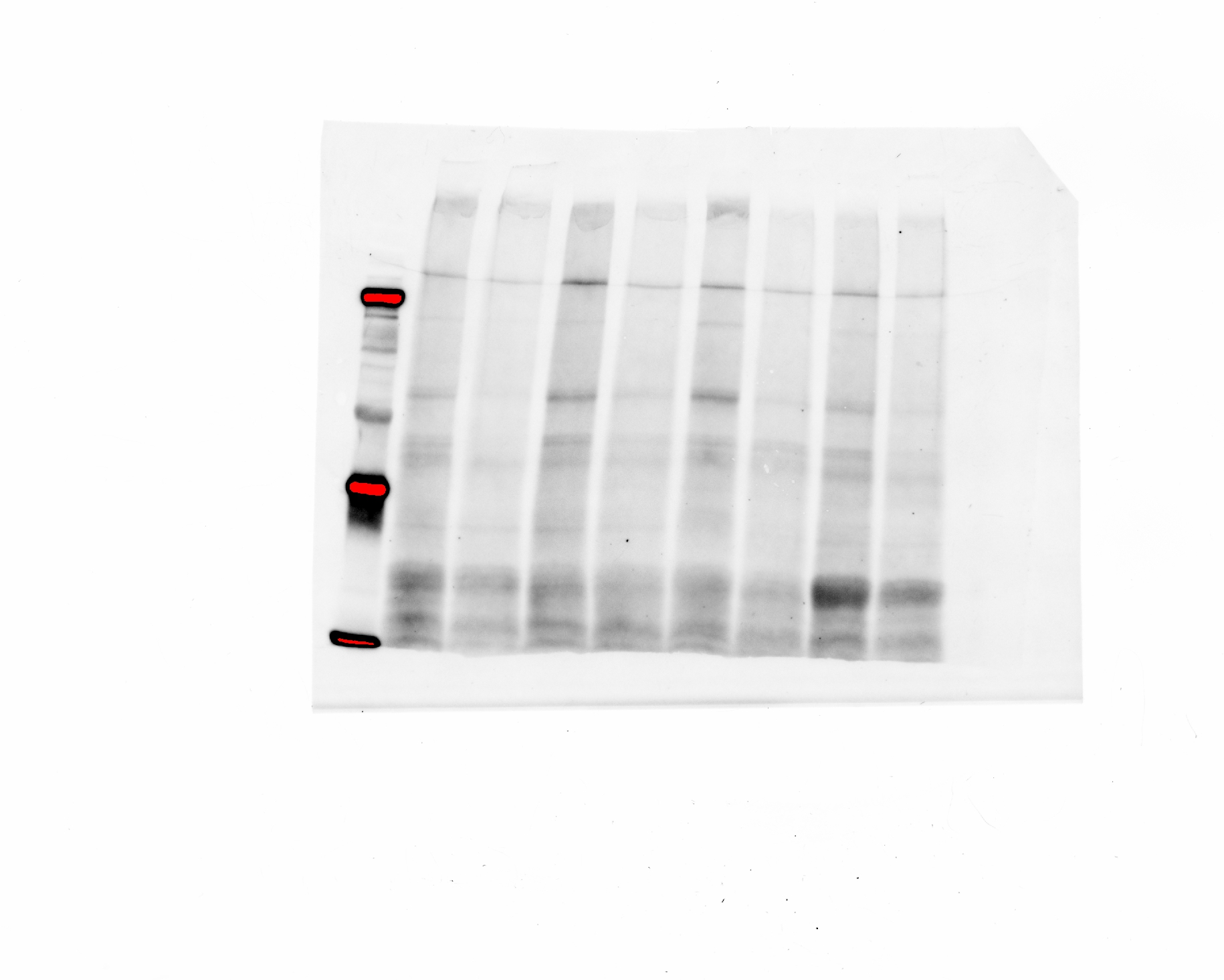

Supplement: Supplementary file 3 — Supplementary Material 3. [file 12964_2024_2015_MOESM3_ESM.zip › non-reducing blot 1 (Stain Free Blot) PRDX5.jpg]

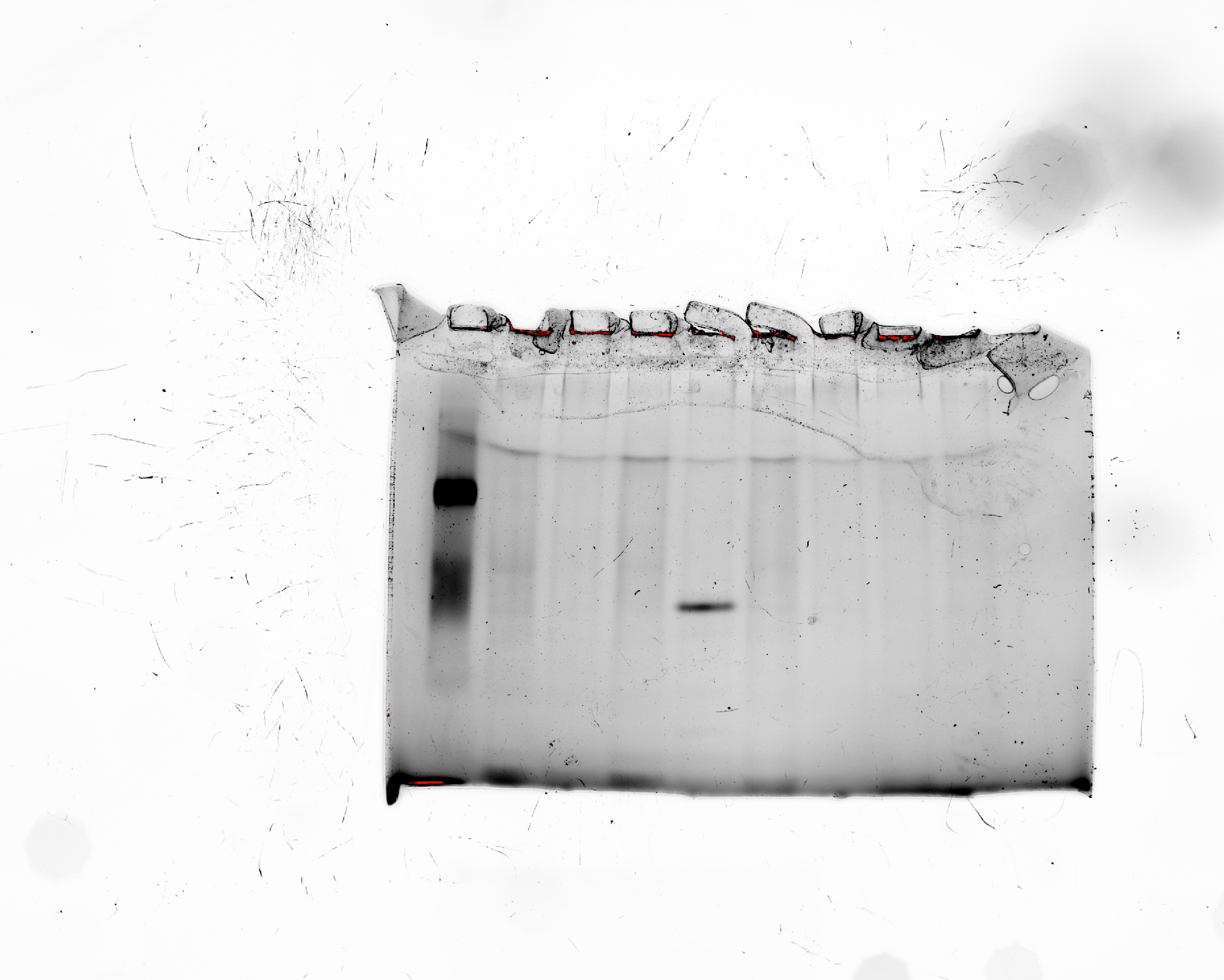

Supplement: Supplementary file 3 — Supplementary Material 3. [file 12964_2024_2015_MOESM3_ESM.zip › PRDX5 -SDS +redukt zel1(Stain Free Gel).tif]

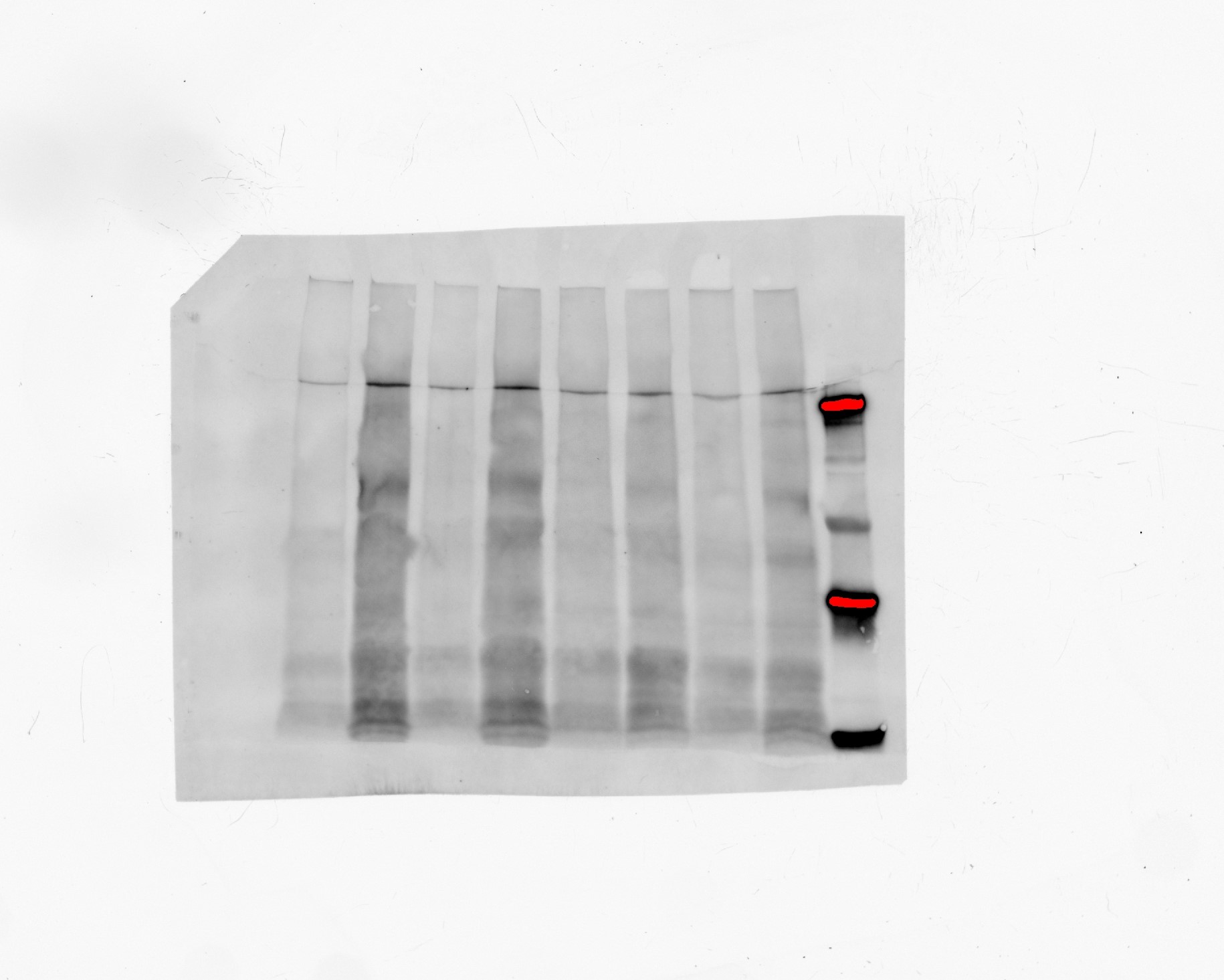

Supplement: Supplementary file 3 — Supplementary Material 3. [file 12964_2024_2015_MOESM3_ESM.zip › PRDX6 non-reducing sw+mroz (Stain Free Blot).jpg]

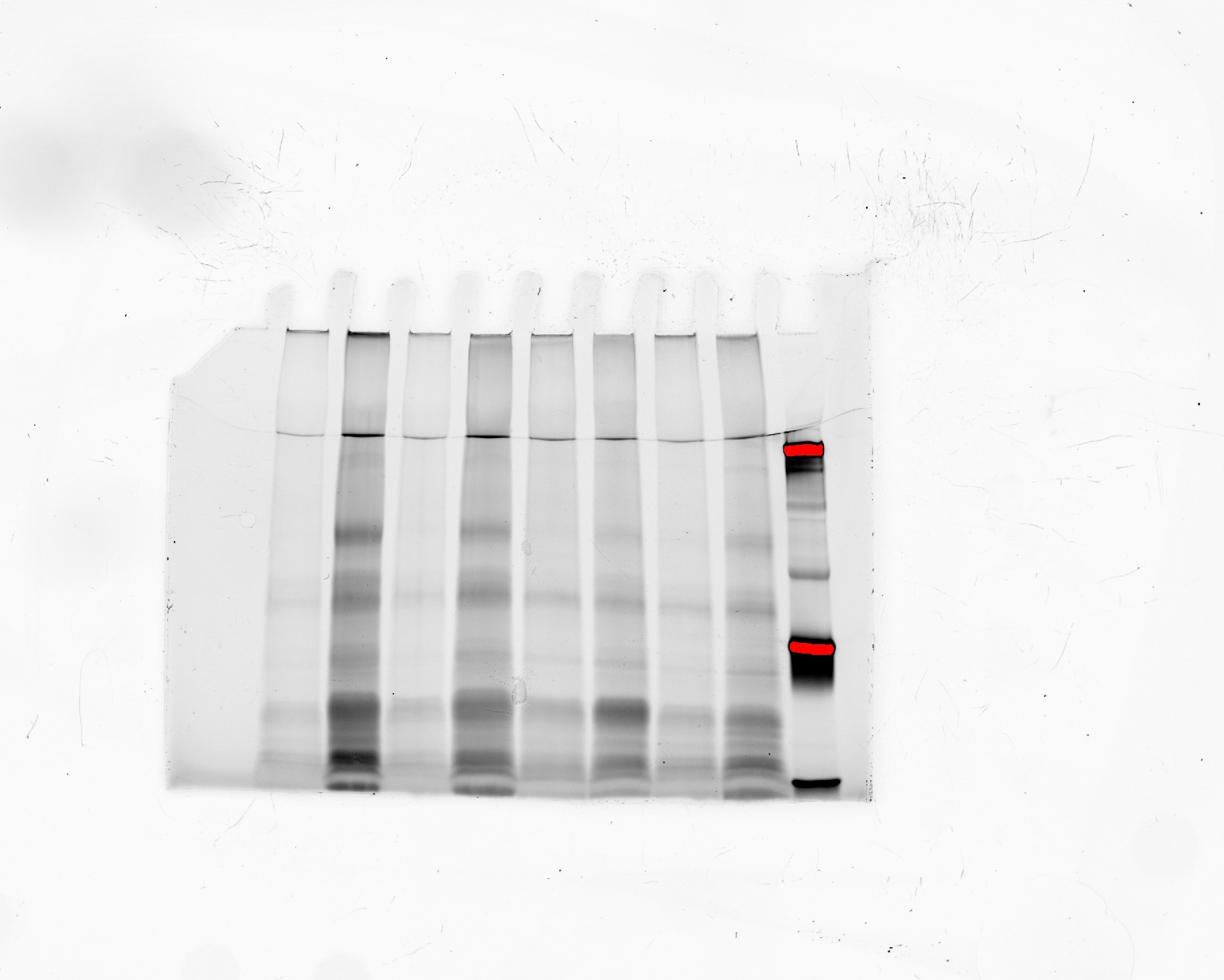

Supplement: Supplementary file 3 — Supplementary Material 3. [file 12964_2024_2015_MOESM3_ESM.zip › PRDX6 non-reducing sw+mroz (Stain Free Gel).jpg]

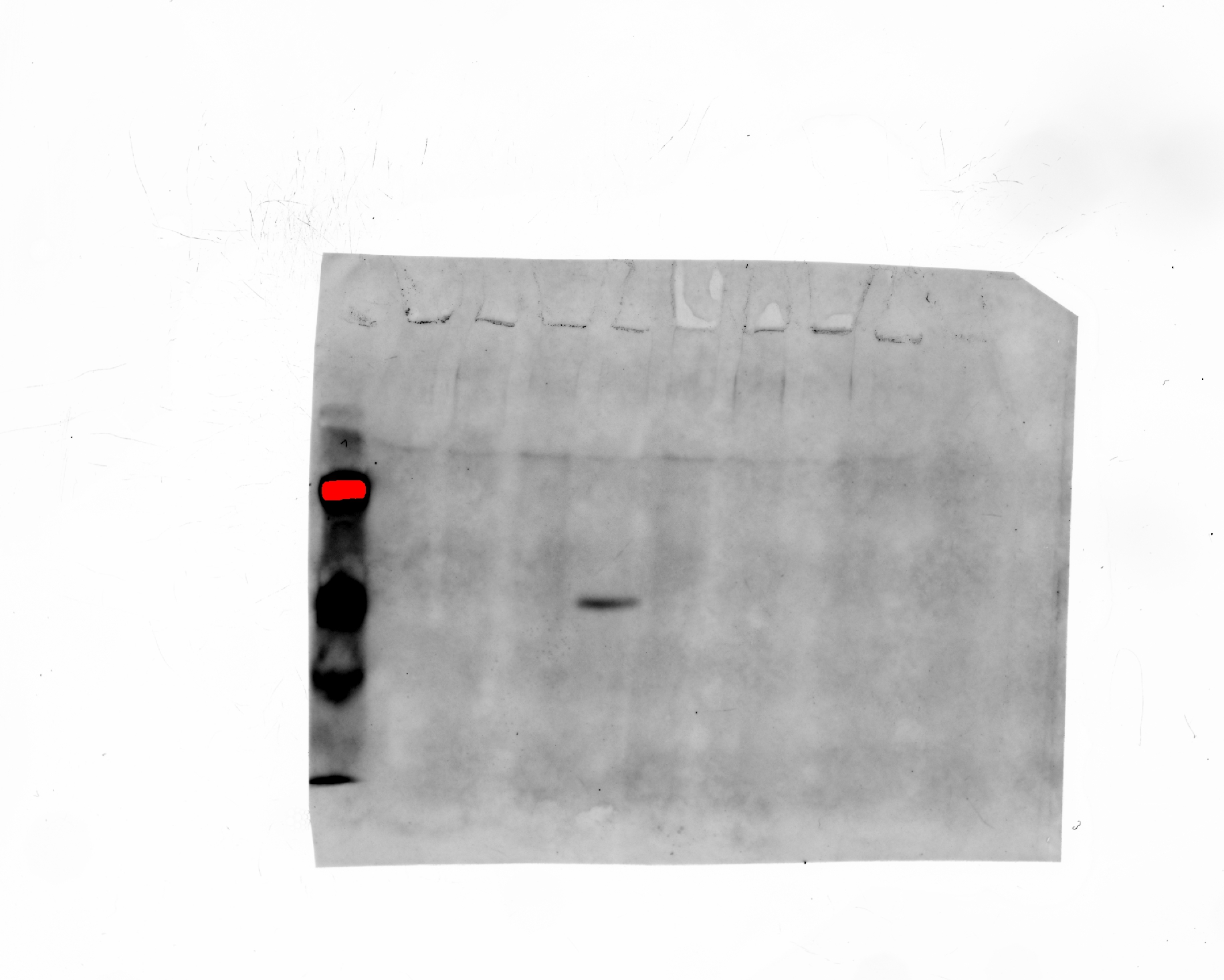

Supplement: Supplementary file 3 — Supplementary Material 3. [file 12964_2024_2015_MOESM3_ESM.zip › -SDS +redukt blot1(Stain Free Blot) PRDX5.jpg]

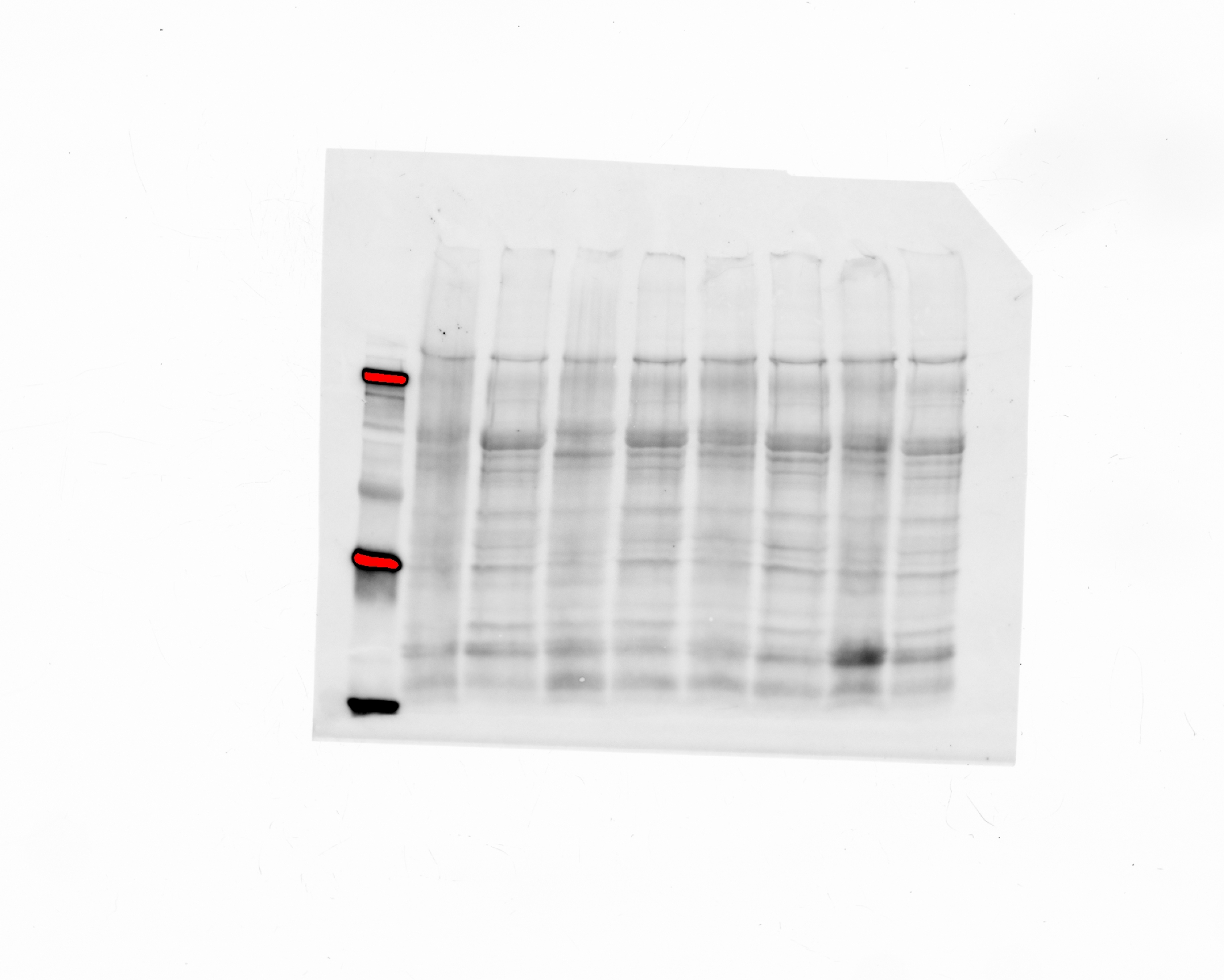

Supplement: Supplementary file 3 — Supplementary Material 3. [file 12964_2024_2015_MOESM3_ESM.zip › standard SDS PAGE blot1(Stain Free Blot) PRDX5.jpg]

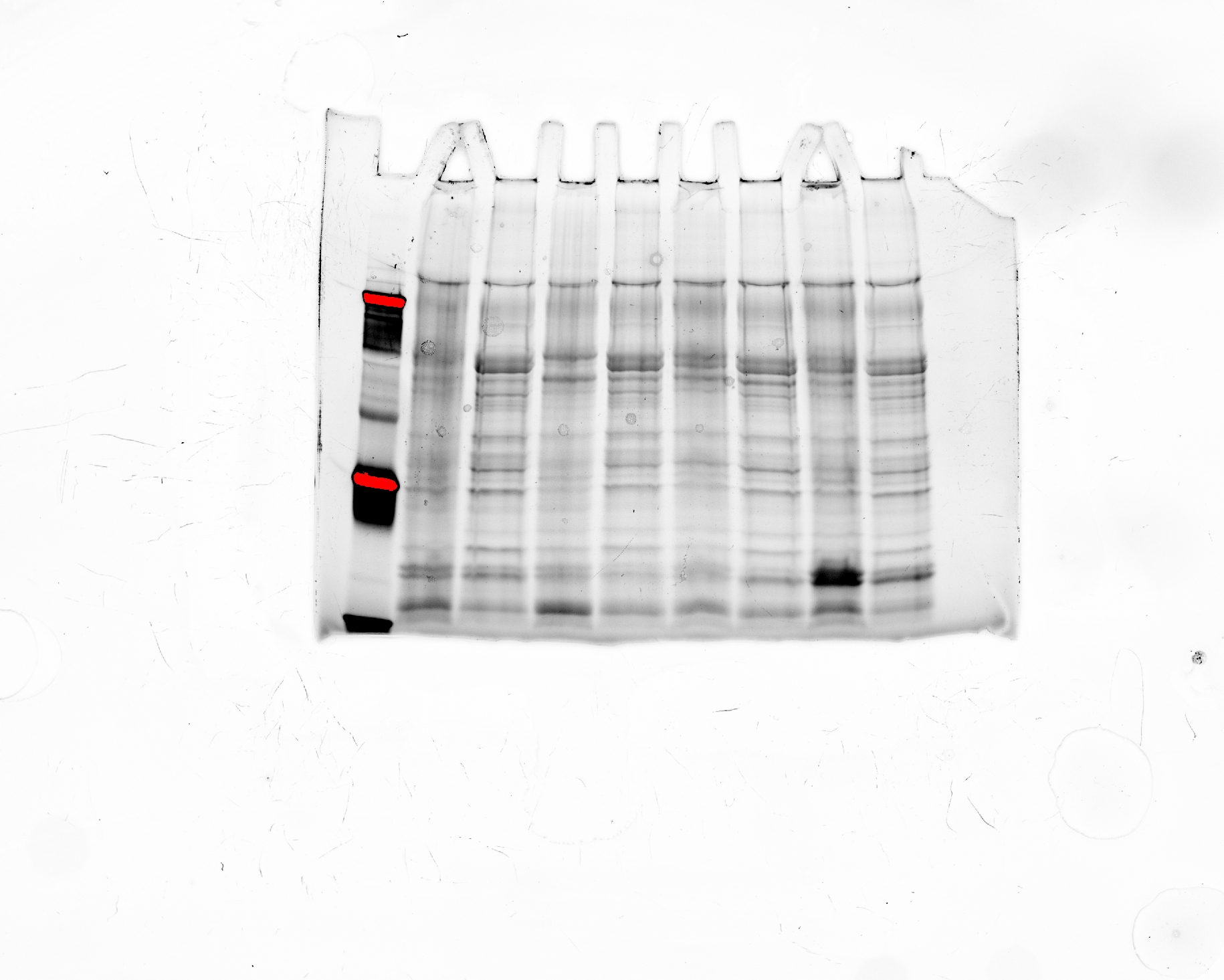

Supplement: Supplementary file 3 — Supplementary Material 3. [file 12964_2024_2015_MOESM3_ESM.zip › standard SDS PAGE zel 1 (stain free gel) PRDX5.jpg]

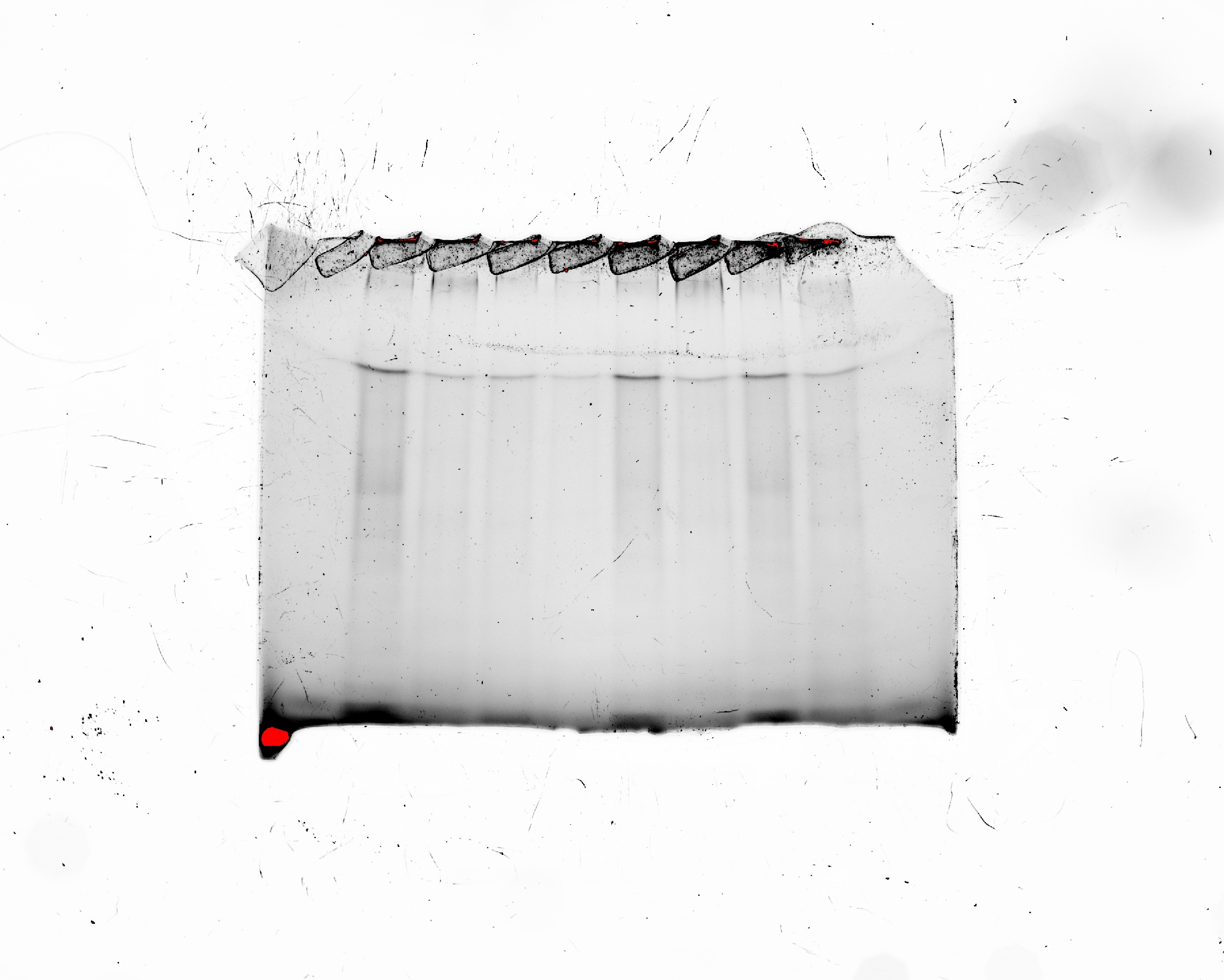

Supplement: Supplementary file 3 — Supplementary Material 3. [file 12964_2024_2015_MOESM3_ESM.zip › zel 1 (-SDS +redukt) (Stain Free Gel) PRDX6.jpg]

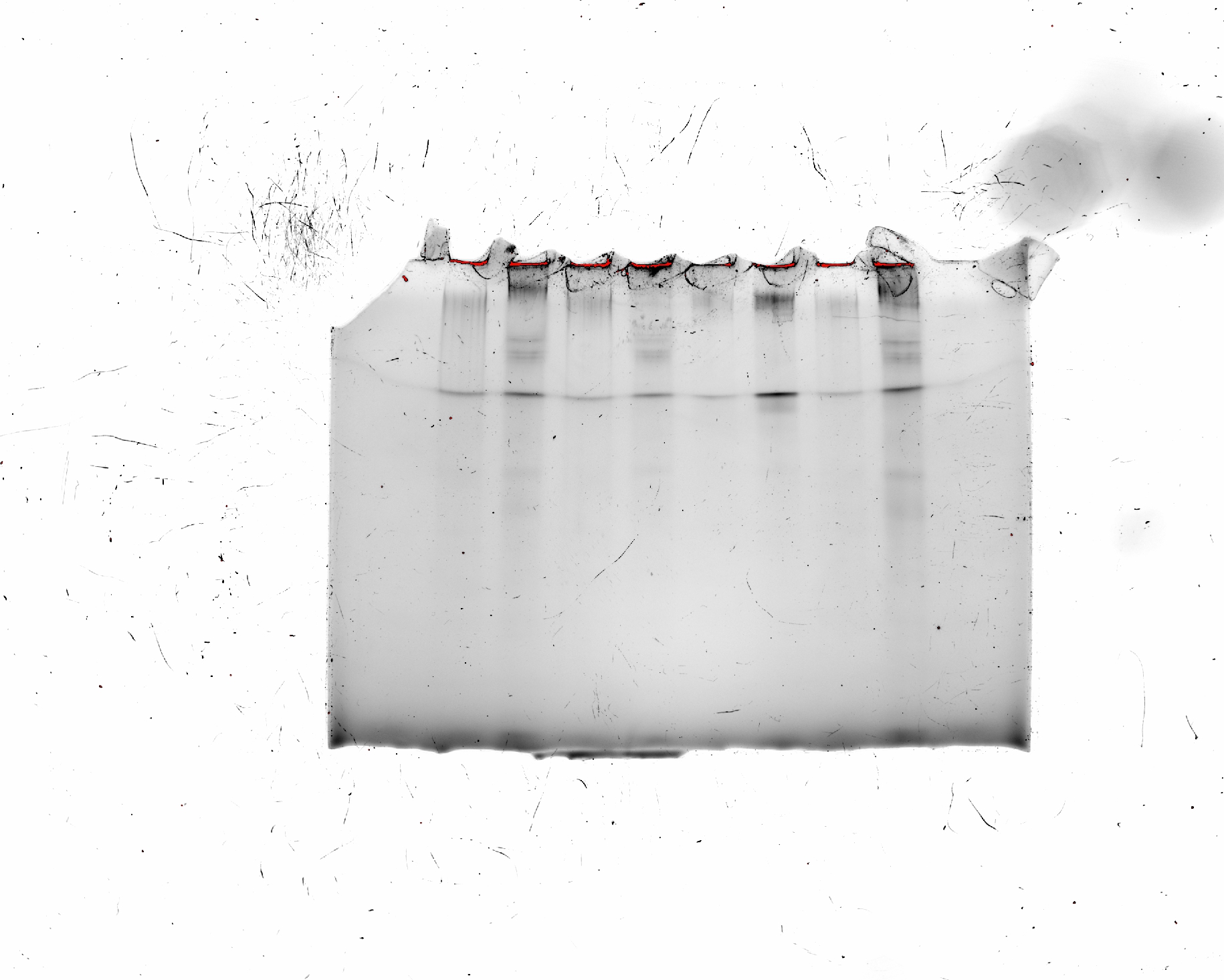

Supplement: Supplementary file 3 — Supplementary Material 3. [file 12964_2024_2015_MOESM3_ESM.zip › zel 1 (-SDS -redukt) (Stain Free Gel) PRDX6.jpg]

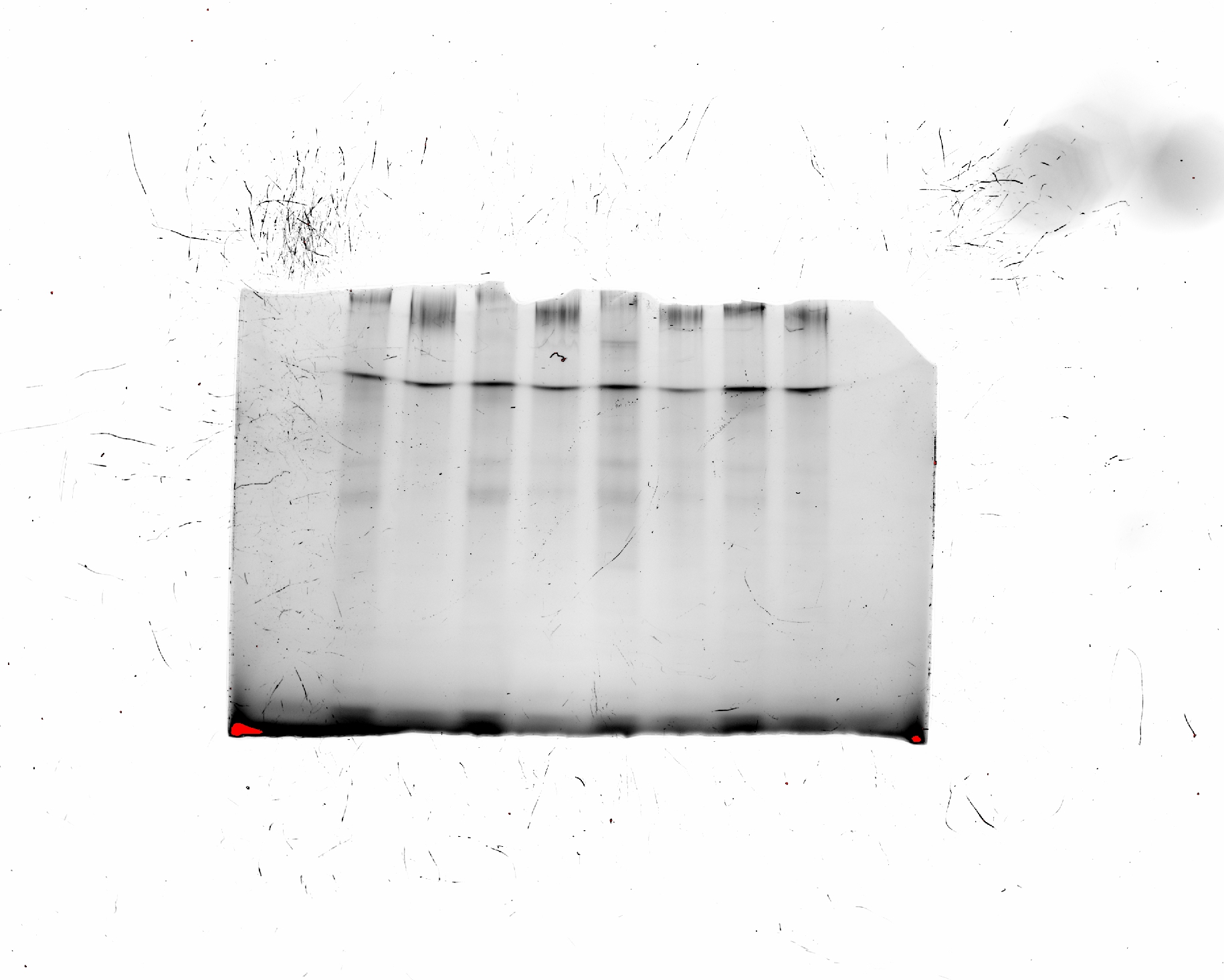

Supplement: Supplementary file 3 — Supplementary Material 3. [file 12964_2024_2015_MOESM3_ESM.zip › zel 1 (stain free gel) PRDX5 native.jpg]
